# Sustainable phytoprotection: a smart monitoring and recommendation framework using Puma Optimization for potato pathogen detection

**DOI:** 10.3389/fpls.2025.1615038

**Published:** 2025-08-15

**Authors:** Amal H. Alharbi, Faris H. Rizk, Khaled Sh. Gaber, Marwa M. Eid, El-Sayed M. El-kenawy, Pushan Kumar Dutta, Doaa Sami Khafaga

**Affiliations:** ^1^ Department of Computer Sciences, College of Computer and Information Sciences, Princess Nourah bint Abdulrahman University, Riyadh, Saudi Arabia; ^2^ Department of Communications and Electronics, Delta Higher Institute of Engineering and Technology, Mansoura, Egypt; ^3^ Computer Science and Intelligent Systems Research Center, Blacksburg, VA, United States; ^4^ Faculty of Artificial Intelligence, Delta University for Science and Technology, Mansoura, Egypt; ^5^ Jadara Research Center, Jadara University, Irbid, Jordan; ^6^ Department of Programming, School of Information and Communications Technology (ICT), Bahrain Polytechnic, Isa Town, Bahrain; ^7^ Applied Science Research Center, Applied Science Private University, Amman, Jordan; ^8^ School of Engineering and Technology, Amity University, Kolkata, India

**Keywords:** precision agriculture, potato disease classification, Restricted Boltzmann Machine (RBM), Puma Optimization algorithm (PO), ecological machine learning

## Abstract

Ensuring sustainable and resilient agricultural systems in the face of intensifying crop disease threats requires intelligent, data-driven tools for early detection and intervention. This study proposes a novel hybrid framework for potato disease classification that integrates copula-based dependency modeling with a Restricted Boltzmann Machine (RBM), further enhanced through hyperparameter tuning using the biologically inspired Puma Optimization (PO) algorithm. The system is trained and evaluated on a real-world dataset derived from structured field experiments, comprising 52 instances and 42 agronomic, microbial, and ecological variables. By fusing copulabased transformations with PO-driven optimization, the framework effectively models complex nonlinear dependencies among heterogeneous features, enabling high-fidelity probabilistic inference in high-dimensional ecological spaces. The RBM baseline outperformed conventional classifiers such as KNN, Random Forest, XGBoost, and MLP, achieving 94.77% accuracy. With PO-based optimization, performance improved significantly to 98.54% accuracy, with parallel gains in sensitivity, specificity, and F1-score. Statistical analysis using ANOVA and Wilcoxon signed-rank testing confirmed the significance of these improvements (p <0.002). In contrast, convergence analysis demonstrated PO-RBM’s computational efficiency relative to PSO, GWO, and GA alternatives. These findings underscore the utility of the proposed framework as a scalable and ecologically grounded decision-support system for integrated pest management (IPM), offering a practical path toward low-impact, adaptive plant health monitoring solutions.

## 1 Introduction

The escalating global demand for food security and environmental protection underscores the urgent need for highly sustainable methods of plant protection Ayilara et al. (2023). The widely cultivated potato plant (*Solanum tuberosum*) faces significant threats to its productivity and quality due to a broad spectrum of diseases, making it particularly vulnerable Okagu et al. (2023). Severe disease outbreaks in potato crops can lead to substantial yield losses, compelling farmers to rely on intensive chemical treatments that degrade soil health, disrupt ecosystems, and threaten biodiversity and human well-being. To align with sustainability objectives, it is imperative to minimize reactive pesticide use by ensuring rapid and accurate disease identification Markam et al. (2024).

Early-stage detection of plant diseases empowers farmers to implement targeted interventions, thereby reducing dependence on agrochemicals while promoting environmentally responsible agricultural practices Elbasi et al. (2023). Such precision in detection renders prophylactic pesticide applications unnecessary, enabling performance-driven disease response strategies that safeguard natural ecosystems. Data-driven technologies are a cornerstone in this transformation Ngugi et al. (2024). Machine learning (ML), in particular, bridges agronomic science with data analytics and systems ecology, effectively integrating complex datasets to uncover patterns for early-warning systems and decision-support tools. Through ML, the intricate high-dimensional interactions among crop variables, soil patterns, and microbial information become interpretable, exposing latent disease risks that conventional monitoring approaches often overlook Pukrongta et al. (2024).

Despite its promise, deploying machine learning in eco-friendly agricultural disease classification encounters numerous technical and ecological challenges Kaur et al. (2023). Potato crop diseases stem not from isolated causes but from a confluence of biological indicators, including microbial diversity, clade richness and evenness, yield dynamics, and localized environmental patterns Vázquez-Ramírez et al. (2023). These factors exhibit complex statistical dependencies, necessitating sophisticated modeling frameworks that capture non-linear behaviors without overspecializing to particular field conditions Yang et al. (2023).

A significant challenge lies in the high dimensionality of input data. The fusion of biological and agronomic indicators for potato disease classification leads to a vast and often redundant feature space—comprising multi-threshold clade richness/diversity metrics, yield variance measures, and nutrient profiles Shaheed et al. (2023). Such redundancy conceals critical data patterns and inflates the computational cost of model training. Accurate and efficient disease classification thus calls for advanced learning architectures and fine-tuned hyperparameters to mitigate instability and ensure consistent error distributions Cheng et al. (2024).

This study introduces a comprehensive predictive system designed to enhance the reliability of potato disease classification by leveraging the capabilities of Restricted Boltzmann Machines (RBMs) in conjunction with Puma Optimization (PO), a biologically inspired metaheuristic algorithm Gijsbers et al. (2021). The PO algorithm optimizes the RBM’s hyperparameters, improving training stability and generalization across heterogeneous field datasets. Unlike traditional static pipelines that rely on heuristicbased parameter tuning Pradhan et al. (2021), the proposed metaheuristic approach performs adaptive searches for optimal configurations that align with the data structure Qu et al. (2024).

The primary objective of this research is to develop an environmentally responsible machine learning framework that achieves technical rigor in disease classification tasks Jha et al. (2024). The methodology incorporates rigorous model evaluation and training optimization tailored to ecological datasets, enhancing the effectiveness of intelligent agricultural monitoring systems. The predictive tools developed herein aim to support farmers, agricultural advisors, and policymakers in making timely decisions for sustainable crop management. The findings substantiate the viability of data-intensive strategies for building resilient and ecologically sound food systems, a priority area in agricultural sustainability and environmental science Xing and Wang (2024).

Limitations hinder current approaches to potato disease classification in adaptability, ecological relevance, and the ability to model complex, nonlinear dependencies in high-dimensional agricultural datasets. Existing models often rely on static hyperparameter tuning and fail to generalize across varying environmental conditions. There is a pressing need for a dynamic and probabilistically grounded framework that captures latent ecological interactions and self-optimizes them in response to diverse and noisy data. This research addresses this gap by integrating copula-based dependency modeling with Restricted Boltzmann Machines, further enhanced via Puma Optimization, to construct an interpretable, scalable, and accurate predictive system for sustainable disease surveillance in precision agriculture.

The key contributions of this work are summarized as follows:

- We introduce a novel hybrid machine learning framework that synergistically integrates copula-based dependency modeling with a probabilistic Restricted Boltzmann Machine (RBM), enabling robust capture of complex, nonlinear, and latent interactions among ecological, microbial, and agronomic variables in high-dimensional agroecosystem datasets.
- We employ the biologically inspired Puma Optimization (PO) algorithm for hyperparameter tuning of the RBM. PO’s adaptive phase-switching behavior dynamically balances exploration and exploitation, significantly enhancing training stability, convergence speed, and classification accuracy compared to conventional optimization methods.
- We develop a comprehensive preprocessing pipeline tailored for heterogeneous ecological data, incorporating missing value imputation (via distribution-aware strategies), Z-score normalization, and log transformations. This design maintains statistical integrity while maximizing model readiness without discarding features.
- We conduct rigorous empirical evaluations of our PO-RBM framework against multiple optimizationenhanced RBM variants (PSO-RBM, GWO-RBM, GA-RBM) and standard classifiers (KNN, Random Forest, MLP, and XGBoost). Our framework consistently achieves superior performance across accuracy, sensitivity, specificity, and F1-score metrics, as validated by statistical tests (ANOVA and Wilcoxon signed-rank).
- We contextualize our approach within sustainable agriculture and integrated pest management (IPM), demonstrating its ecological interpretability, practical utility for real-time decision-making, and scalability for deployment in data-driven crop disease surveillance systems.

This research paper consists of the following sections: Section 2 surveys recent potato disease detection methods while examining classification technologies and agricultural monitoring solutions that utilize machine learning. The research describes its materials and methods, which consist of dataset processing methods alongside dependency models based on copulas and machine learning algorithms with metaheuristic optimization approaches. Section 4 contains the descriptive evaluation metrics results and experimental findings from baseline and optimized configuration models. The research presents findings under Section 5 while focusing on three essential points about sustainable agriculture, model robustness, and ecological relevance of the study results. Section 6 of this study presents the conclusions, including significant research contributions and potential directions for better adaptive disease monitoring systems in intelligent agroecosystems.

## 2 Related works

Potatoes (*Solanum tuberosum*) represent one of the world’s most essential staple crops, making accurate disease detection vital for food security and sustainable agricultural practices. Numerous machine learning and imaging approaches have been proposed for early disease detection, quality assessment, and crop monitoring. This section categorizes existing research into four major themes—CNN-based visual detection, hyperspectral imaging techniques, UAV-based surveillance systems, and hybrid/spectral optimization frameworks—while critically assessing their contributions, limitations, and unresolved challenges.

### 2.1 CNN-based visual detection

Convolutional Neural Networks (CNNs) have remarkably succeeded in visual disease detection tasks. Marino et al. (2019) adopted a weakly supervised CNN model to detect and segment six classes of potato skin defects. Although the model achieved substantial precision (0.91) and recall (0.90), it lacked integration with agronomic or ecological data, limiting its interpretability for field applications.

(Agarwal et al., 2020) proposed a deep CNN architecture to classify early and late blight in potato leaves under varying environmental conditions, achieving testing accuracy as high as 99.8%. While the approach excels in visual pattern recognition, it relies heavily on image quality and consistency, making it sensitive to field noise.

(Gao et al., 2021) used deep neural networks for lesion segmentation, reporting IoU values of 0.996 for the background class but only 0.386 for disease lesions—highlighting challenges in detecting fine-grained pathology details.

(Hou et al., 2021) used a graph cut algorithm followed by an SVM for leaf disease classification, achieving 97.4% accuracy. Though promising, the system is tailored for controlled settings and does not scale to mixed agronomic data.

Recent advancements in CNN-based disease detection have focused on enhancing model architecture and broadening disease classification scope. Mahum et al. (2023) proposed a novel framework using an improved DenseNet-201 architecture to classify five distinct disease categories, achieving 97.2% accuracy. Chauhan et al. (2023) introduced a CNN-based model that automatically extracts potato leaf features, attaining a precision of 98.9%. Durai et al. (2023) used ResNet50 for image segmentation followed by CNN classification to detect early and late blight, achieving 99.75% accuracy. Most recently, Srivastava et al. (2024), introduced a DenseNet-CNN model trained on a potato leaf database, reaching 99.6% accuracy.

### 2.2 Hyperspectral and multispectral imaging

Hyperspectral imaging (HSI) has emerged as a powerful tool for detecting visible and internal potato defects. Ji et al. (2019) leveraged HSI combined with Multi-class SVM (MSVM) and LDA for dimensionality reduction to classify six potato defect types. This approach achieved up to 90% accuracy but required extensive preprocessing and uniform lighting conditions.

Zhang et al. (2019) employed a multispectral imaging method (676–952 nm) with LS-SVM for real-time potato defect detection, reporting 90.7% accuracy. However, the reliance on specialized spectral hardware can limit scalability. A similar spectral calibration strategy was adopted by Bienkowski et al. (2019), using partial least squares and neural networks to detect symptomatic and pre-symptomatic infections, but with reduced accuracy (74.6%) in field conditions due to environmental variability.

(Yang et al., 2021) introduced a spectral germination detection approach using supervised multiple threshold segmentation (SMTSM) and a Canny edge detector. Although this achieved a high actual positive rate (TPR) of 90.91%, the model’s dependence on multispectral imaging limits its accessibility to resource-constrained farmers.

### 2.3 UAV-based and remote sensing methods

Remote sensing platforms, particularly UAVs, offer scalable disease monitoring. Rodríguez et al. (2021) investigated UAV-derived multispectral imagery with five ML classifiers to detect late blight. Random Forest and LSVM yielded the highest precision. Despite strong results, this method requires radiometric corrections and struggles with occlusion and mixed pixels in real-time scenarios.

Sentinel-2 time series imagery was employed in (Ashourloo et al., 2020) for soil/crop discrimination without ground-truth data. Achieving a kappa coefficient over 0.8, this method introduced novel spectral signatures for potato fields. However, while effective for classification, it lacks precision in disease-type identification and does not incorporate microbial or agronomic complexity.

To synthesize recent advancements in potato disease detection and classification, **Table 1** provides a comparative overview of key studies drawn from the literature. Each entry highlights the modeling techniques employed, the specific agricultural application targeted, and the principal outcomes reported.

**Table 1:** Summary of related works on potato disease detection and classification.

Study	Technique/Model	Application	Key Outcome
(Zhang et al., 2019)	LS-SVM + Multispectral Imaging	Potato defect detection using spectral reflections (676–952 nm)	High contrast image segmentation; 90.7% accuracy
(Ji et al., 2019)	HSI + MSVM + LDA	Potato defect classification using visible-NIR spectrum	Accuracy up to 90%; LDA used for dimensionality reduction
(Marino et al., 2019)	CNN + Weakly Supervised Learning	Flaw detection and quality Classification (6 potato classes)	Achieved 0.91 precision and 0.90 recall on complex dataset
(Bienkowski et al., 2019)	PLS + BPNN	Pre-symptomatic and visible disease identification	84.6% accuracy; 94% lesion classification precision; lower accuracy in field conditions
(Agarwal et al., 2020)	CNN with 4 convolution layers	Detection of early and late blight via leaf images	Achieved over 99% accuracy; handles variable environments
(Ashourloo et al., 2020)	Sentinel-2 Time Series	Soil/crop discrimination without ground-truth	¿90% accuracy; novel potato spectral signatures derived
(Rodríguez et al., 2021)	RF, GBC, SVC, LSVC, KNN	UAV-based late blight detection via ML	Best precision with RF and LSVM; robust image preprocessing pipeline
(Gao et al., 2021)	Deep Neural Networks + Segmentation	Late blight lesion severity quantification using RGB images	IoU 0.996 (background), 0.386 (lesion); useful in breeding
(Hou et al., 2021)	Graph Cut + SVM	Leaf disease and severity classification	IoU: 93.7%; SVM accuracy up to 97.4% (disease) and 91% (severity)
(Yang et al., 2021)	SMTSM + Canny + Genetic Programming	Detection of germination in spectral images	TPR of 90.91%, precision of 89.28%; top performer in detection accuracy
(Durai et al., 2023)	ResNet50 + CNN	Early and late blight detection from segmented leaf images	Achieved 99.75% accuracy; superior to existing models
(Chauhan et al., 2023)	CNN with feature extraction	Detection and classification of potato leaf diseases	Achieved 98.9% precision; automated image-based recognition
(Mahum et al., 2023)	Improved DenseNet201	Five-class potato disease classification leaf	Achieved 97.2% accuracy using balanced, extended dataset
(Srivastava et al., 2024)	DenseNet-CNN	Image-based potato disease detection leaf	Achieved 99.6% accuracy; robust against visual variation

### 2.4 Critical gaps in existing research

The reviewed works underscore substantial progress in potato disease detection using AI and imaging methods. However, several limitations persist:

- Lack of data integration: Most models use visual or spectral data alone, overlooking agronomic and microbial indicators critical in disease onset.
- Fixed or manual hyperparameter tuning: Few systems employ adaptive mechanisms to optimize model parameters, which limits their generalizability across field conditions.
- Ecological interpretability: Black-box models such as deep CNNs offer limited insight into underlying biological processes, reducing their utility for ecological decision-making.
- Over-reliance on controlled datasets: Many models achieve high accuracy in curated environments but degrade in real-world, noisy, and heterogeneous farming contexts.

### 2.5 Comparative advantage of our approach

The current study addresses these limitations through a novel hybrid framework that:

- Integrates copula-based modeling to capture nonlinear dependencies across diverse ecological, microbial, and agronomic variables;
- Employs a Restricted Boltzmann Machine (RBM) for probabilistic representation of highdimensional inputs;
- Applies the Puma Optimization (PO) algorithm to optimize hyperparameters for stable and accurate model convergence dynamically;
- Frames disease classification within the context of sustainable agriculture and environmental monitoring, offering transparency and scalability for real-time, field-deployable decision support systems.

By systematically addressing technical and ecological challenges, our approach contributes a versatile and interpretable tool for precision agriculture and Integrated Pest Management (IPM).

## 3 Materials and methods

In addition to standard optimization techniques like Genetic Algorithm (GA) and Particle Swarm Optimization (PSO), this study employed Puma Optimization (PO), a newer bio-inspired metaheuristic known for its adaptive phase-switching behavior and ability to balance exploration and exploitation in complex search spaces. PO was selected due to its enhanced convergence reliability and proven performance in avoiding premature stagnation—a limitation often encountered with more established algorithms in highdimensional, non-convex problems such as ecological classification. Its dynamic adjustment mechanism made it particularly suitable for tuning the probabilistic parameters of the RBM, where training sensitivity and stability were critical.

**Figure 1:**
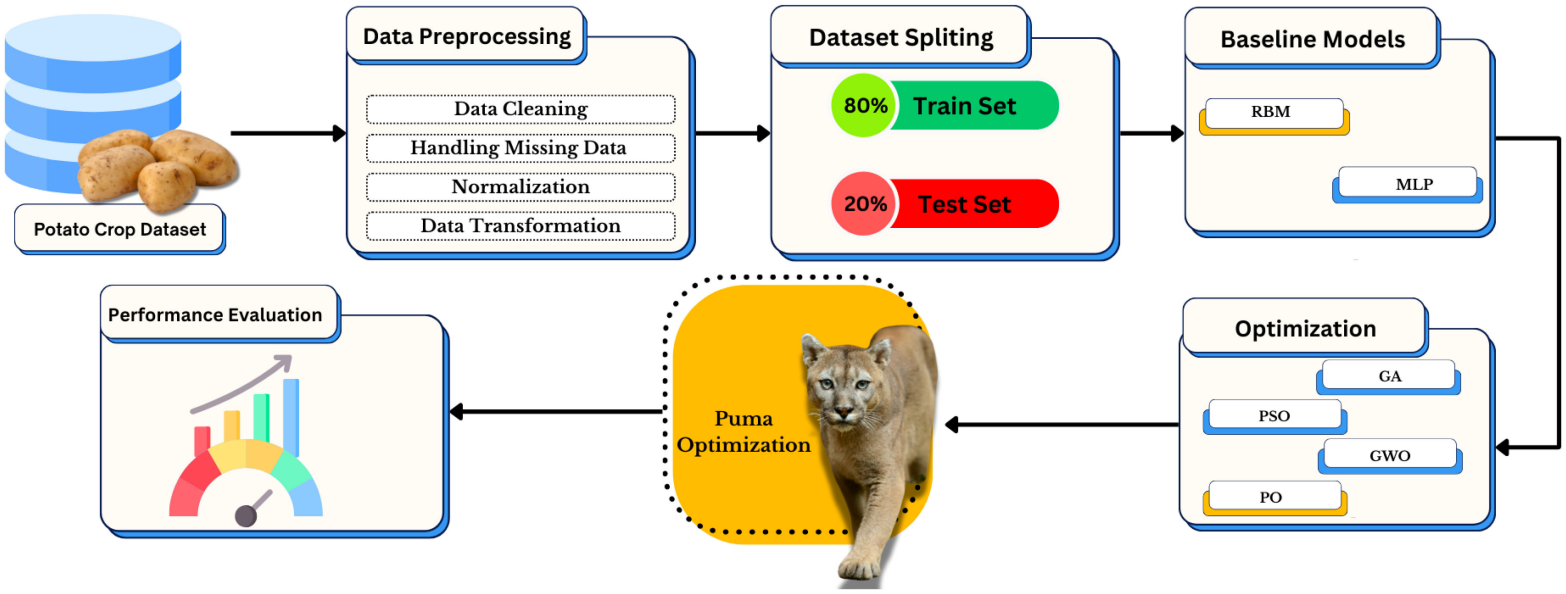
Framework of the proposed approach for potato crop prediction using data preprocessing, feature selection, model training, and performance evaluation.

The complete diagram illustrating the methodology for enhancing potato crop yield prediction methods can be found in **Figure 1**. A systematic data processing sequence begins with pre-treating a potato crop dataset by cleaning data, resolving missing values, and normalizing and transforming it before proceeding. The research adopts Restricted Boltzmann Machine (RBM) and Multi-Layer Perceptron (MLP) as baseline models after training-test data splitting. The selection process depends on multiple metaheuristic algorithms such as Genetic Algorithm (GA), Particle Swarm Optimization (PSO), Grey Wolf Optimizer (GWO), and Puma Optimization (PO), where the last method enhances performance additionally. Performance testing of the model occurs at the end of the framework to identify the most suitable configuration.

### 3.1 Dataset overview

The dataset used in this study comprises 52 samples collected from structured field experiments conducted across 26 distinct potato cultivation sites, each identified by a unique FieldID. These sites span multiple environmental and management conditions, contributing to ecological diversity in the data. The experiments were replicated across two experimental seasons, denoted by the Experiment identifier, to account for temporal variability in soil conditions, microbial communities, and crop stress factors. Specifically, the dataset encompasses 26 unique fields × 2 experiments = 52 instances, ensuring balanced temporal and spatial sampling coverage.

The field trials were conducted in Wisconsin, USA, as part of the potato microbiome research efforts coordinated by the Solís-Lemus Lab. Sampling took place during the spring and summer seasons, which align with the active growth and disease development phases for potato crops in this region. This seasonal and geographic context enhances the dataset’s representativeness for real-world potato disease dynamics in temperate agricultural zones.

Each instance in the dataset includes a comprehensive set of agronomic indicators, microbial diversity metrics, and nutrient variables. These features characterize yield behavior, ecological stress, and potential disease susceptibility. The binary classification target, PD, denotes disease presence (PD = 1) or absence (PD = 0) based on field symptom assessments.

The distribution of PD values is stratified across a continuous range of observed disease severity scores. Notably, each severity level appears exactly twice, confirming a balanced representation across the sample set. This design ensures that no particular disease condition dominates the learning process, promoting unbiased model generalization.

The data were stratified into training and testing subsets to prepare for predictive modeling while preserving the distribution of disease and non-disease samples. This stratified split was performed at the instance level rather than the field level to maintain environmental representation across both partitions.

Given the relatively small dataset size, a noise injection strategy was adopted to mitigate overfitting and enhance generalization. Gaussian noise (mean = 0, standard deviation = 5% of each variable’s standard deviation) was added to numerical features grouped by FieldID and nutrients. This data augmentation technique preserved original statistical properties while increasing the diversity of training instances without affecting categorical or label variables.

Moreover, to further reduce overfitting risks, dropout regularization was applied to the visible layer of the Restricted Boltzmann Machine (RBM) during training. A dropout rate of 0.3 was used, which randomly zeroes out 30% of the inputs during each forward pass, thus preventing co-adaptation of hidden units.

A tabular summary of the dataset features is presented in **Table 2**, detailing the feature names, types, and short descriptions to improve reproducibility.

**Table 2:** Summary of dataset features.

Feature Name	Type	Unit/Scale	Description
FieldID	Categorical	N/A	Identifier for the field site
Experiment	Categorical	N/A	Identifier for the experimental season
yields_average	Numeric	Yield units	Average potato yield from a plot
yields_variance	Numeric	Yield units^2^	Variability in yield values
yields_inverse.var	Numeric	1/Yield units^2^	Inverse of yield variance, indicating stability
yields_std.err. mean	Numeric	Yield units	Standard error of the mean yield
seCSscore	Numeric	Arbitrary units	Composite score of yield and environmental features
claderichX	Numeric	Richness Index	Clade richness at various diversity thresholds ( X=0.10 to 0.80)
cladedivX	Numeric	Diversity Index	Clade diversity at various thresholds ( X=0.10 to 0.80)
asvrich	Numeric	Count	Amplicon sequence variant (ASV) richness
asvdiv	Numeric	Index	ASV diversity
nutrients	Numeric	Arbitrary units	Composite index of nutrient availability
PD	Numeric (Binary)	0 or 1	Disease presence label (1 = diseased, 0 = healthy)

### 3.2 Data preprocessing

The extensive heterogeneity and complexity of the dataset needed an essential data preprocessing phase to proceed with the methodological pipeline. Field and laboratory observation data formed the original dataset which exhibited missing values among features and incompatible measurement scales and had different levels of measurement accuracy. Multiple cleaning steps were established to establish the method.

The preprocessing pipeline began with detecting missing values before applying specific imputation tactics depending on the respective feature characteristics. The mean was used for yield-related features when the variable exhibited near-normal distribution. At the same time, the median was selected for variables with mild skewness or visible outliers to minimize the influence of extreme values. The procedure replaced missing values in yield averages and variances by applying mean or median substitution according to empirical distribution patterns derived from histograms and skewness diagnostics. For microbial diversity indices, missing values were relatively rare and occurred in continuous variables with smooth trends across samples; therefore, interpolation was used to preserve ecological continuity and avoid biasing richness or diversity estimates.

The normalization process applied to all numerical features came after imputation. The normalization step standardized input variable ranges so large-scale features could not dominate the training process. A Z-score normalization process sets the mean to zero and the standardized deviation to one for all variables. Such a normalization approach proved beneficial because it accommodated the mixture of both low-scoring diversity measurements and yield metrics with high variance.

The analysis included the application of data transformation techniques to improve linearity as well as decrease skewness characteristics. Logarithmic transformations were applied to variance-based metrics with long-tailed distributions to achieve Gaussian approximation. The modification allowed models to converge better while conforming to prevailing assumptions that natural systems operate on log-normal distributions.

The dataset received a final check for multicollinearity and redundancy issues. The structure of RBM models required assessment of input dependence, but this process needed further development through copula modeling and hyperparameter optimization approaches. The preprocessing step created a uniform statistical framework that enabled better ecological interpretation and improved computational efficiency for upscaling procedures in the following modeling tasks.

In addition to preprocessing, the RBM model employed L2 regularization with a weight decay factor of $1\times 10^{-4}$ to prevent overfitting by penalizing large weight values during training. This, combined with dropout and augmentation, formed a multi-pronged regularization strategy suitable for the small sample size.

**Figure 2:**
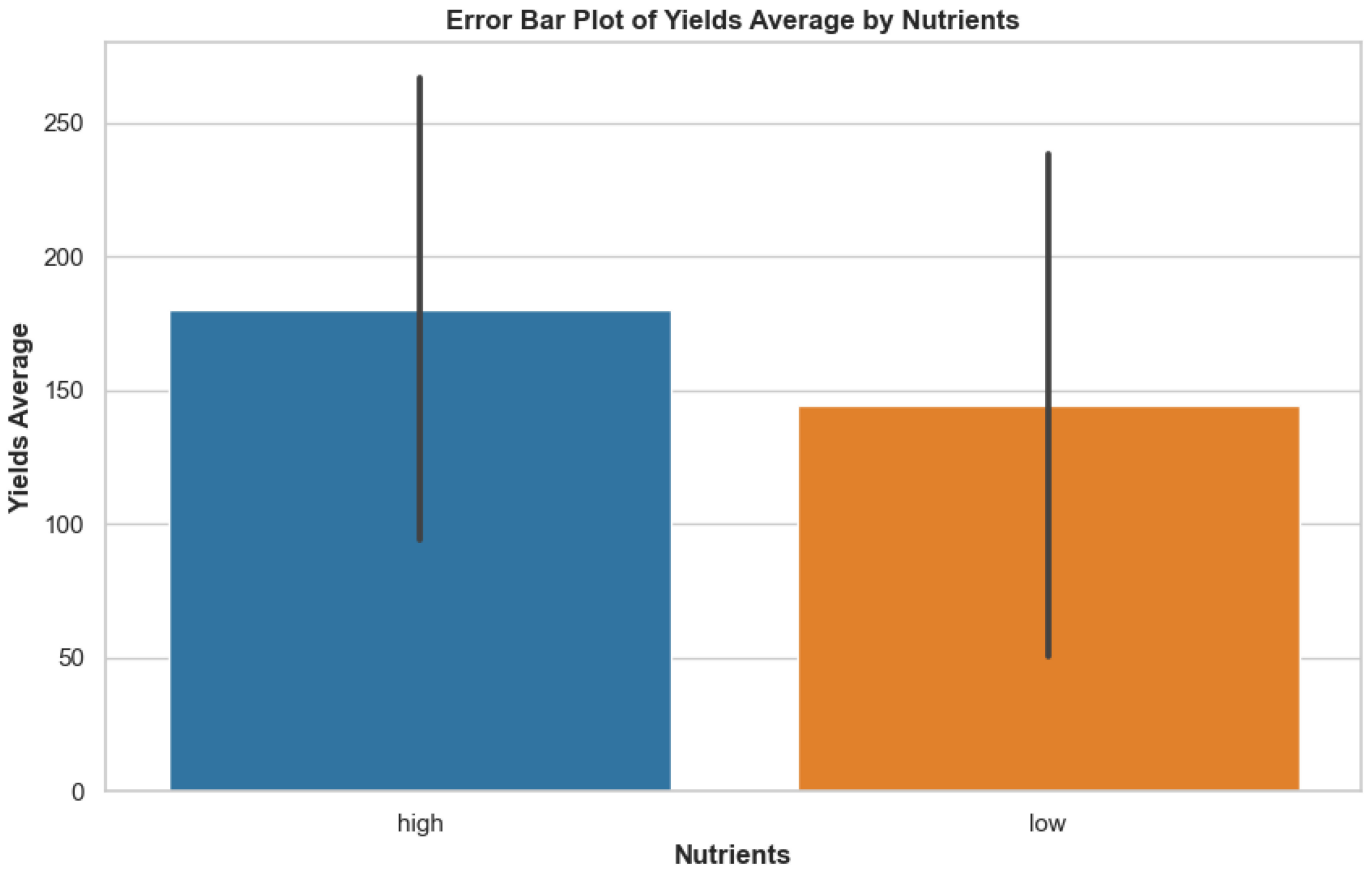
Error bar plot showing the average yields under high and low nutrient conditions, with standard deviation represented as error bars.

The constructed error bar graphic showcases yield average differences between high and low nutrient conditions. The visualization depicted in **Figure 2** shows the variation and central point data regarding yield measurements across different nutrient conditions, which reveals how nutrient accessibility shapes potato crop productiveness. This is useful for identifying statistically meaningful gaps in yield performance across environmental conditions, providing ecological context for model interpretation.

**Figure 3:**
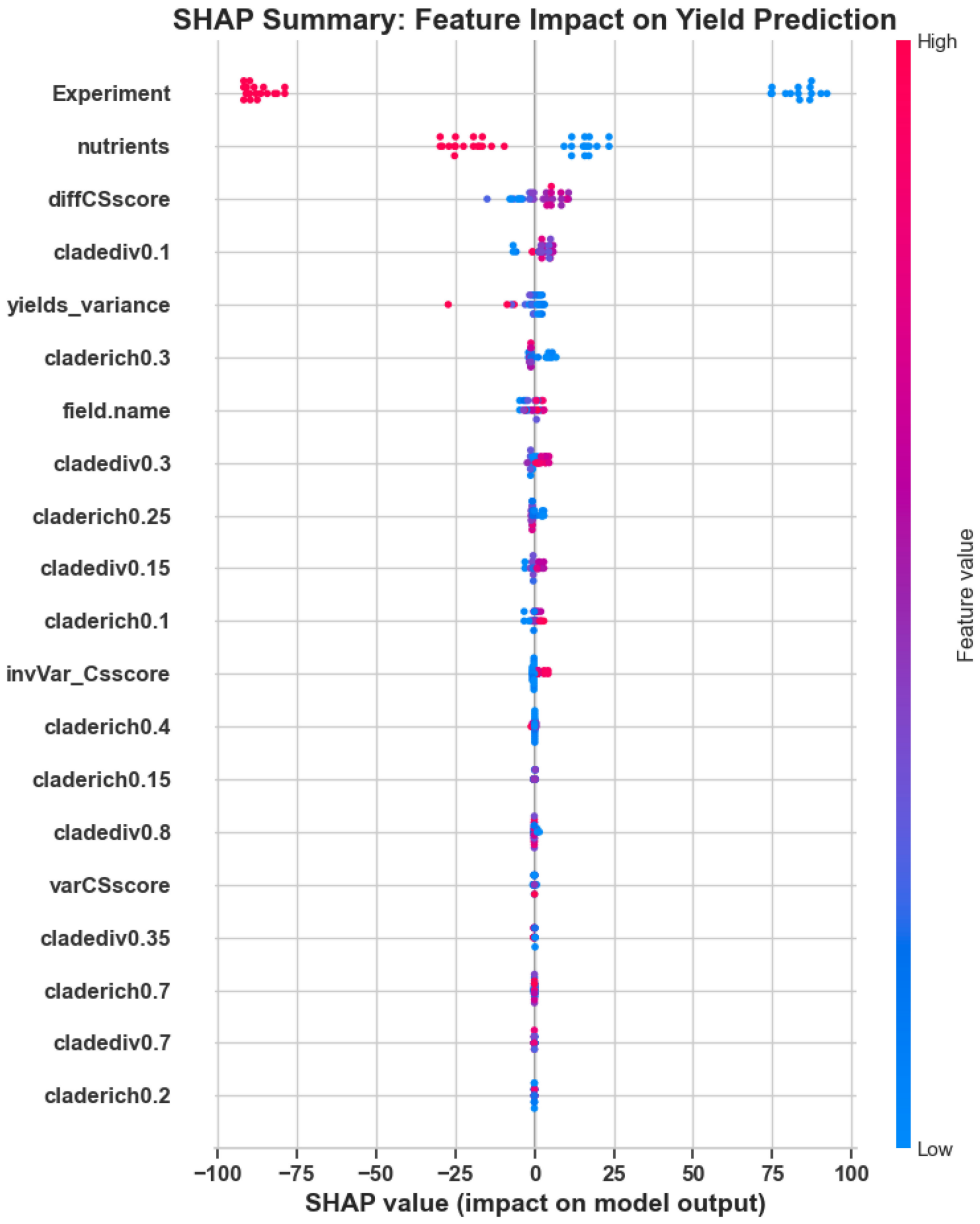
SHAP summary plot: Feature-wise distribution of SHAP values illustrating each variable’s contribution to yield prediction. Higher SHAP values indicate greater influence on model output.

To validate the model’s ecological interpretability, we performed a SHAP (SHapley Additive exPlanations) analysis to assess the contribution of each feature to yield prediction outcomes. **Figure 3** illustrates the distribution of SHAP values for the most influential features, with Experiment, nutrients, and diffCSscore emerging as key drivers of model behavior.

**Figure 4:**
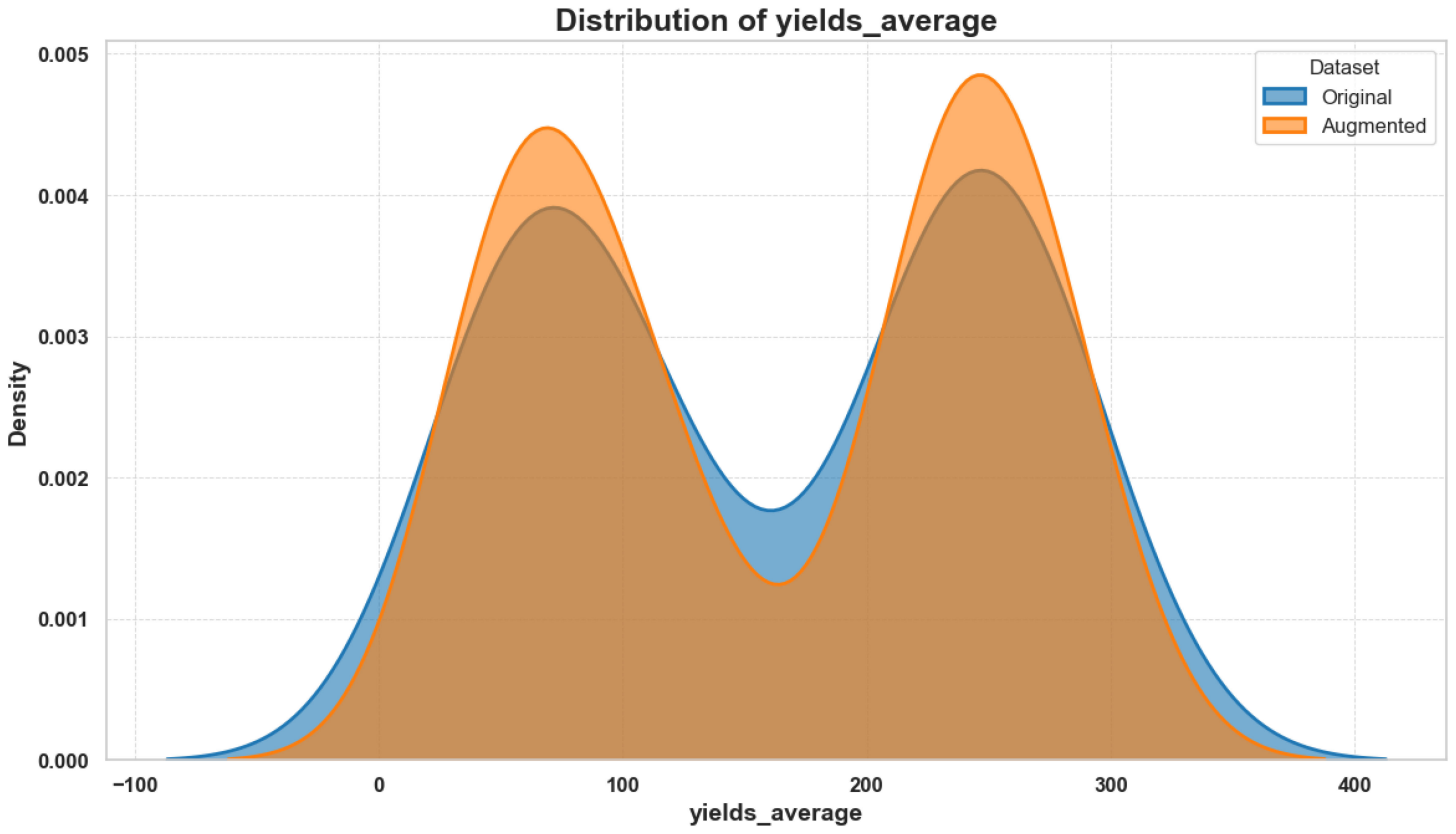
Top SHAP feature importances: Bar chart showing average absolute SHAP values for the most impactful ecological variables.

To complement the distributional view, **Figure 4** ranks features by their mean absolute SHAP impact. This bar chart confirms that ecological and agronomic parameters—particularly nutrients and microbial diversity indices such as cladediv0.1 and yields_variance—carry meaningful interpretability in the context of disease risk modeling.

### 3.3 Copula-based dependency modeling

In ecological and agro-environmental data analysis, the traditional correlation approaches fail to capture distinct complex non-Gaussian relationships between variables because they possess nonlinear patterns Joe (2014). Organizations dealing with high-resolution biological metrics alongside agronomic and physiological indicators face this issue in their datasets. The joint analysis of diverse variables with copula-based models achieves accurate statistical detection due to its ability to preserve single variable distributions during dependency assessment.

A copula is a multivariate distribution function that enables decomposing a joint probability distribution into its marginal distributions and a dependence structure. Formally, given a multivariate random vector **X** = (*X*_1_ *,X*_2_ *,…,X_n_*), with marginal cumulative distribution functions (CDFs) *F*_1_ *,F*_2_ *,…,F_n_*, Sklar’s Theorem ensures the existence of a copula *C* such that their joint CDF *F* can be expressed as:


(1) $$F(x_{1},x_{2},\ldots ,x_{n})=C(F_{1}(x_{1}),F_{2}(x_{2}),\ldots ,F_{n}(x_{n})).$$

This formulation allows the modeling of marginal behavior independently from the dependencies among variables, making copulas especially suitable for environmental applications where variable distributions may differ substantially in scale, shape, and support.

The research utilized copulas to monitor and evaluate the associations between various features within the potato disease dataset. These features encompass yield variability, microbial diversity metrics at multiple taxonomic thresholds, and nutrient concentrations—each with distinct statistical properties and biological interpretations. Converting multiple variables into uniform scales through their marginal CDFs enables them to be connected using copula functions, revealing hidden ecological structures that traditional linear correlation matrices cannot detect.

This research gained particular value from copula modeling because of its two essential applications. The study benefited from using copula modeling to detect non-linear and asymmetric relationships between research variables, including the impact of clade diversity at thresholds in the range (e.g., cladediv0.35) on yield variations and disease risks despite low linear correlations. The standardized probabilistic representation of data created through copula-based transformation made the model-experienced feature set more compatible with the probabilistic learning in Restricted Boltzmann Machines.

In contrast to simple normalization—which only scales individual variables—or correlation matrices—which capture only linear dependence, copula modeling revealed conditional dependencies that improved feature interactions within the learning architecture. For example, clade diversity at 0.35 and nutrient stress indicators showed negligible Pearson correlation but demonstrated strong joint behavior under disease conditions when modeled with copulas. This enabled the RBM to form more meaningful hidden representations, significantly boosting classification accuracy after optimization.

Using copulas matches ecological modeling standards because they enable scientists to identify predictive joint distribution patterns of community responses to environmental stressors. Agro-ecosystems experience disease onset when multiple factors affect microbial community imbalance, nutrient deficiencies, and physiological stress, driving disease dynamics. Copula framework delivers better ecological process modeling by directly handling joint probability distributions between multiple variables.

The present study employed copula-based dependency modeling for twofold purposes: first, to generate ecological knowledge through preanalytic modeling and second, to transform data so it could optimize the efficiency of subsequent machine learning algorithms. The pipeline incorporates this model, demonstrating dedication to statistical precision and environmental representation, boosting the utility of interdisciplinary solutions for plant disease prevention systems.

**Figure 5:**
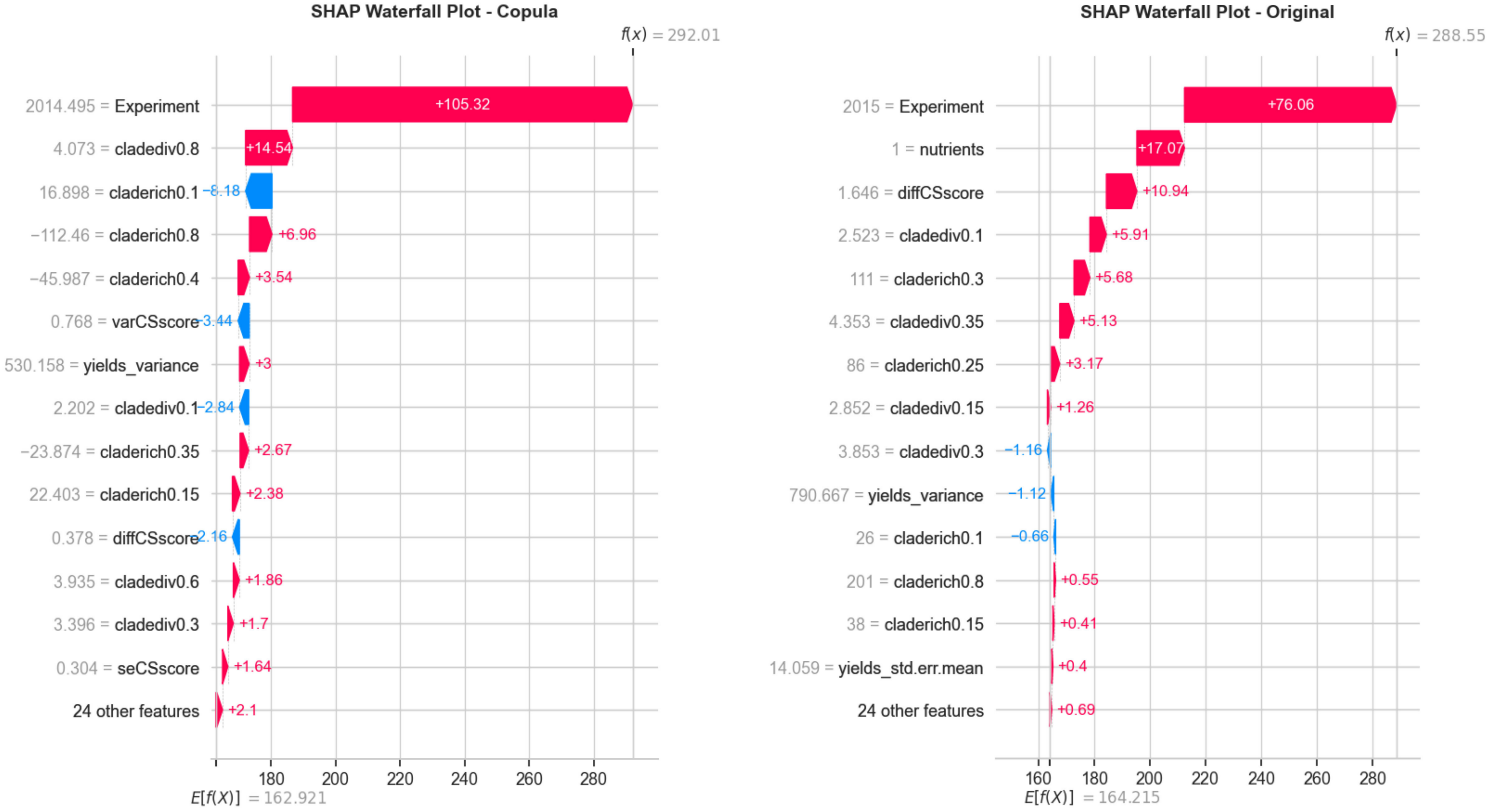
Side-by-side visualization of (Left) pairwise comparison between original and copula-generated features and (Right) SHAP waterfall plot showing the key ecological contributors to a representative prediction from the original dataset. The combination confirms structural fidelity and interpretability of the modeling pipeline.

To evaluate how well the copula-generated data aligns with the statistical structure of the original dataset, we conducted a pairwise feature distribution comparison and SHAP interpretability analysis. **Figure 5** presents these results side by side. On the left, the overlay of original and copula-generated values for key ecological variables demonstrates close alignment in density and scatter patterns, confirming that the copula model preserves joint distribution characteristics. On the right, a SHAP waterfall plot for the original dataset reveals the most influential features in a representative prediction, highlighting ecological variables such as Experiment, nutrients, and diffCSscore.

**Figure 6:**
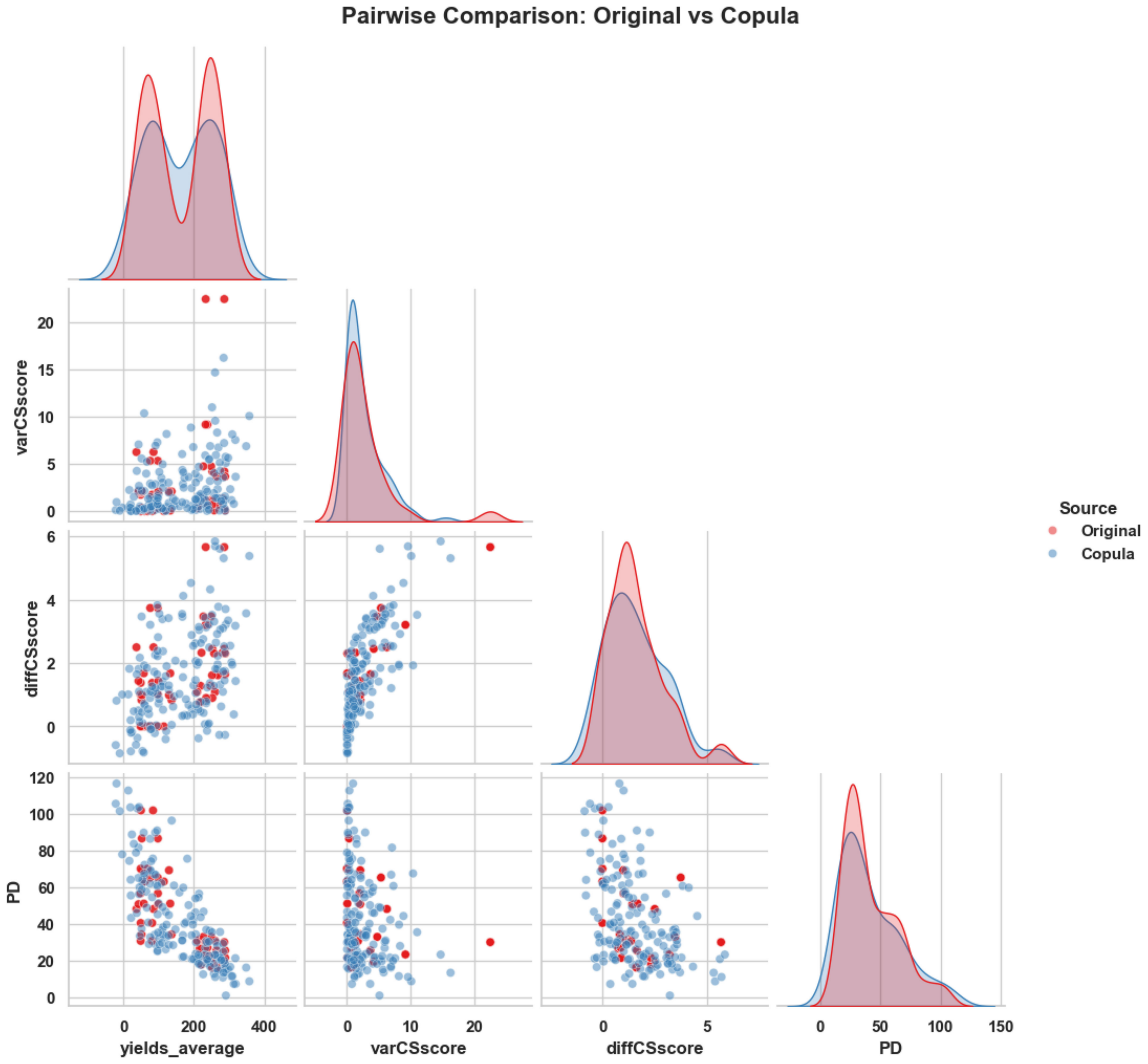
SHAP waterfall plot — copula-generated data: Confirms the same influential ecological factors as seen in the original dataset, reinforcing the interpretability of the model under data augmentation.

In comparison, the SHAP waterfall plot for the copula-augmented dataset (**Figure 6**) demonstrates similarly ranked influential features, with slight variation in magnitude. This affirms the ecological relevance retained through synthetic data transformation and validates that PO-RBM remains interpretable even with simulated inputs.

### 3.4 Machine learning models

This study implemented two machine learning models based on multivariate ecological and agronomic features to predict potato disease occurrence: the Restricted Boltzmann Machine (RBM) and the MultiLayer Perceptron (MLP). The RBM served as the core predictive architecture due to its ability to model hidden, nonlinear relationships in high-dimensional data. At the same time, the MLP was used as a baseline for comparative evaluation.

#### 3.4.1 Restricted Boltzmann Machine

An RBM is a generative stochastic neural network comprising two layers: a visible layer **v** ∈ ℝ*^n^* and a hidden layer **h** ∈ ℝ*^m^*, with full inter-layer connections and no intra-layer connections Fischer and Igel (2012). It models the joint probability distribution of inputs via an energy function:


(2) $$E(\text{v},\text{h})=-\sum_{i}a_{i}v_{i}-\sum_{j}b_{j}h_{j}-\sum_{i,j}v_{i}W_{ij}h_{j},$$

where *W_ij_* are the weights and *a_i_,b_j_* are the biases. The probability distribution is:


(3) $$P(\text{v},\text{h})=\frac{1}{Z}e^{-E(v,h)},$$

with *Z* as the partition function. RBMs are typically trained via contrastive divergence to approximate the true data distribution.

RBMs are well-suited to agroecological data because they can learn hidden structures from complex, highdimensional, and partially overlapping variables. This study trained a baseline RBM using preprocessed input data and fixed hyperparameters (e.g., hidden units, learning rate, batch size). Its performance served as a benchmark for subsequent optimization using metaheuristics.

The RBM was selected over more conventional classifiers such as Support Vector Machines (SVMs) and Random Forests (RF) because it offers a probabilistic and generative learning framework that can model latent interactions in ecological data—particularly useful when relationships among variables are nonlinear and not easily separable in feature space. Unlike RF or SVMs, which primarily rely on surface-level feature splits or margin optimization, RBMs can uncover hidden patterns between microbial diversity, yield stability, and nutrient availability contributing to disease outcomes. This generative aspect complements the unsupervised structure of ecological signals, making RBMs a fitting choice for this complex classification task.

#### 3.4.2 Used benchmark machine learning models

To provide a comprehensive performance baseline, four commonly used classifiers were included in the benchmarking process: K-Nearest Neighbors (KNN) Guo et al. (2003), Random Forest (RF) Breiman (2001), Multi-Layer Perceptron (MLP) Popescu et al. (2009), and XGBoost Chen and Guestrin (2016).

KNN is a non-parametric, instance-based learning algorithm that classifies samples based on the majority label among the *k* nearest neighbors in feature space. While interpretable and straightforward, KNN can be sensitive to feature scaling and high-dimensional noise.

RF is an ensemble-based decision tree model that builds multiple randomized trees and aggregates their predictions through majority voting. It is robust to overfitting and performs well in many structured data applications, including ecological modeling.

MLP is a supervised feedforward neural network with multiple hidden layers with nonlinear activation functions. It learns complex patterns in data using backpropagation and is commonly used for structured tabular data, although it lacks the generative capabilities of RBMs.

XGBoost is a gradient-boosting framework optimized for performance and speed. It builds decision trees sequentially by minimizing a loss function, offering strong regularization and efficiency, especially in medium to large datasets.

All benchmark models were trained and evaluated using the same preprocessing pipeline and stratified data partitioning to ensure fair performance comparisons under consistent experimental conditions. Their inclusion allows for evaluating the relative strengths of the RBM architecture within a diverse set of learning paradigms.

### 3.5 Metaheuristic optimization

#### 3.5.1 Optimization objective

Excessive disease incidence modeling in potato crops requires precise machine-learning algorithm setups due to diverse conditions combined with complex, high-dimensional, and noisy data. The Restricted Boltzmann Machine (RBM) stands out through its powerful functionality alongside sensitivity, which requires precise adjustment of its learning rate, number of hidden units, and momentum and weight initialization parameters for correct operation. The model’s performance in learning ecological relations and data generalization depends strongly on proper parameter setting across all its hyperparameters.

The hyperparameter tuning methods are based on traditional grid search or random search operations inefficiently and rigidly while working with high-dimensional hyperparameter spaces. These methods lack the flexibility to respond appropriately when handling the probabilistic relationships observed within ecological data because variable interactions change significantly between different environmental settings.

The research implements state-of-the-art metaheuristic optimization methodologies as a solution for training RBMs. Metaheuristics work excellently for complex optimization problems lacking gradient information, making them suitable for RBM model tuning despite non-convex error landscapes and stochastic behavior. The main goal of metaheuristic implementation in this research is finding the best suitable RBM hyperparameter combinations, which lead to high classification results while minimizing variance throughout multiple runs to achieve accurate and reliable predictions.

#### 3.5.2 Proposed optimizer: Puma Optimization

Puma Optimization (PO) represents the optimizer of choice in this study, while Abdollahzadeh et al. (2024) originally developed it. The Puma Optimization algorithm shows how pumas (*Puma concolor*) as territorial predators manage their hunting patterns by combining exploration of new prey areas with hunting in existing productive zones. Based on past search performance feedback, the phase-switching mechanism uses an ecological model that transforms between exploratory and exploitative behaviors Abdollahzadeh et al. (2024).

At each iteration *t*, the position of a candidate solution ${\vec{X}}_{i}^{t}$ in the search space is updated based on one of two behaviors:


(4) $${\vec{X}}_{i}^{t+1}=\{\begin{array}{ll} {\vec{X}}_{i}^{t}+{\vec{r}}_{1}\cdot N(0,1)\cdot ({\vec{X}}_{\text{best}}^{t}-{\vec{X}}_{i}^{t}) & \text{if exploration}\\ {\vec{X}}_{i}^{t}+{\vec{r}}_{2}\cdot \text{cos }(\theta )\cdot ({\vec{X}}_{i}^{t}-{\vec{X}}_{\text{worst}}^{t}) & \text{if exploitation} \end{array}$$

Here, ${\vec{X}}_{\text{best}}^{t}$ and ${\vec{X}}_{\text{worst}}^{t}$ represent the best and worst solutions in the population at time *t*, ${\vec{r}}_{1}$ and ${\vec{r}}_{2}$ are random vectors sampled from uniform distributions in [0,1], and N (0,1) denotes a standard normal distribution. The cosine term cos(*θ*), with *θ* representing a random angular deviation, introduces an oscillatory adjustment pattern that emulates real-world path curvature observed in territorial puma movement.

Crucially, the algorithm employs a scoring function Ψ to determine whether the next update should be exploratory or exploitative:


(5) Ψ=α·INT+β·RES+γ·DIV

This expression defines INT (intensification) to evaluate solution quality improvements in the most recently generated solutions. At the same time, RES (resonance) detects consistent solution improvements, and DIV (diversity) tracks novel solutions in the current population. The three components are jointly balanced by the tunable coefficients *α,β,γ* ∈ [0,1]. The scoring system enables the algorithm to operate as a hyper-heuristic controller, which allows adaptation based on past and current feedback suitability when navigating complex searching spaces like the ecological fitness landscapes.

The pseudo-code of the PO algorithm used in our research appears in **Algorithm 1** to help researchers understand and replicate the process. This code demonstrates all the optimizer’s vital functions, from initialization to the adaptive phase-switching process. The algorithm combines exploratory and exploitative strategies through random perturbations and strategic updates, drawing inspiration from puma behavioral characteristics.

Algorithm 1 **Puma optimization algorithm (PO).**


1: **Input:** Objective Function *f*(*x*), where *x* = [*x*
_1_
*,x*
_2_
*,…,x_d_
*]
2: **Input:** Population size *N_pop_
*, maximum iterations *MaxIter*
3: **Input:** Control parameters: *PF*
_1_
*,PF*
_2_
*,PF*
_3_
*,U,L,a*
4: **Initialize** population *X_i_
*, *i* = 1*,…,N_pop_
*randomly within bounds [*lb,ub*]
5: **Evaluate** fitness of all *X_i_
*, and identify the best solution as *Puma*
_male_
6: **for** *iter* = 1 to *MaxIter* **do**
7:   **if** *iter* ≤ 3 **then**
8:     **Perform both exploration and exploitation:**
9:     **for** each candidate *X_i_
*
**do**
10:       Generate random number *rand*
_1_
11:       **if** *rand*
_1_ *>* 0.5 **then**
12:         *Z_i,G_
*← *RDim* · (*ub* − *lb*) + *lb*
13:       **else**
14:         Generate *Z_i,G_
*using the prescribed update (see Eq. (25) in PO paper)
15:       **end if**
16:       **for** each dimension *j* **do**
17:         **if** *j* = *j_rand_
*or *rand*
_3_ *< U* **then**
18:           *X*
_new_[*j*] ← *Z_i,G_
*[*j*]
19:         **else**
20:           *X*
_new_[*j*] ← *X_i_
*[*j*]
21:         **end if**
22:       **end for**
23:       Evaluate *f*(*X*
_new_)
24:       **if** *f*(*X*
_new_) *< f*(*X_i_
*) **then**
25:         Update *X_i_
*← *X*
_new_
26:         Update *U* according to Eqs. (28)–(30)
27:       **end if**
28:     **end for**
29:     **Repeat for the exploitation phase using the corresponding strategy (cf. Eq. (32))**
30:     Update phase scores *f*
_1_, *f*
_2_
31:   **else**
32:     Compute scores *f*
_1_, *f*
_2_, *f*
_3_ for both phases
33:     Compute *F*
_explore_ and *F*
_exploit_ using Eqs. (19)–(20)
34:     **if** *F*exploit *>F*explore **then**
35:       **Perfor m exploitation phase:**
36:       **for** each *X_i_
*
**do**
37:         Generate random number *rand*
_4_
38:         **if** *rand*
_4_ *<* 0.5 **then**
39:           **if** *rand*
_5_ *<* 0.5 **then**
40:             Use short-sprint strategy (cf. Eq. (32), case 1)
41:           **else**
42:             **if** *rand*
_6_ *< L* **then**
43:               Use ambush strategy (cf. Eq. (32), case 2a)
44:             **else**
45:               Use long-jump strategy (cf. Eq. (32), case 2b)
46:            **end if**
47:         **end if**
48:       **end if**
49:       Evaluate *f*(*X*
_new_)
50:       if *f*(*X*
_new_) *< f*(*X_i_
*) then
51:         Update *X_i_
*← *X*
_new_
52:       **end if**
53:     **end for**
54:     **else**
55:       **Perform exploration phase (as in lines 7–20)**
56:     **end if**
57:   **end if**
58:   **for** each *X_i_
*
**do**
59:     **if** *f*(*X_i_
*) *< f*(*Puma*
_male_) **then**
60:       Update *Puma*
_male_ ← *X_i_
*
61:     **end if**
62:   **end for**
63: **end for**
64: **Output:** Best solution *Puma*
_male_ and its fitness

RBM configurations receive fitness assessments through training data classification accuracy while the algorithm continuously improves the hyperparameter set to obtain maximum objective results. This research implements the PO algorithm because tests have proven that it excels over conventional metaheuristics in complex optimization problems while keeping diverse populations and preventing premature stopping.

#### 3.5.3 Comparative optimizers

A comparative analysis of PO is required using three established metaheuristic benchmarks, namely *Particle Swarm Optimization* (PSO), *Grey Wolf Optimizer* (GWO), and *Genetic Algorithm* (GA). The optimizers were chosen among those most frequently used in computational ecology because they demonstrate different algorithm design approaches and fulfill RBM training’s stochastic optimization prerequisites.

Within PSO, the algorithm demonstrates swarm behavior by allowing agents to adjust their flight paths through their best locations and the positions of partnering agents Kennedy and Eberhart (1995). The algorithm balances searching for new possibilities and capitalizing on discovered opportunities, giving it wide versatility. GWO draws its inspiration from how grey wolves behave while hunting as a group in natural environments Mirjalili et al. (2014). The algorithm stands out because it shows superior convergence characteristics and a simple design. The GA depends on evolutionary fundamentals of selection, crossover and mutation to build its candidate answer search via successive iterations Reeves (2010). The maximum strength of Genetic Algorithms occurs from their capacity to create wide-ranging solution sets over time, but this process demands extended execution periods.

The comparative algorithms were deployed with default parameters into an RBM training framework that processed PO. The uniform testing approach let researchers isolate the effects of search quality from variations that stemmed from either coding variations or surrounding environmental conditions.

The metaheuristic optimization solution of this research marks an essential methodological advancement to boost accuracy and stability and improve the ecological validity of disease classification in potato cultivation. The research significantly advances sustainable agricultural intelligence through its novel biological PO algorithm, which executes systematic benchmark technique evaluation to establish a robust optimization method for the field.

### 3.6 Evaluation metrics

Multiple performance measures are used to evaluate classification models alongside metaheuristic optimization of the hyperparameters. The metrics deliver a complete performance evaluation that merges accurate disease forecast results with correctly identifying healthy instances. Environmental decisionmaking based on disease detection depends on sensitive, accurate, favorable rates and specific, accurate negative rates as metrics in ecological monitoring.

**Table 3** summarizes the equations for the key evaluation metrics used in this study:

**Table 3:** Evaluation metrics equations.

Metric	Equation
Accuracy	$\frac{TP+TN}{TP+TN+FP+FN}$
Sensitivity (TPR)	$\frac{TP}{TP+FN}$
Specificity (TNR)	$\frac{TN}{TN+FP}$
Positive Predictive Value (PPV)	$\frac{TP}{TP+FP}$
Negative Predictive Value (NPV)	$\frac{TN}{TN+FN}$
F1-Score	$\frac{2TP}{2TP+FP+FN}$

The count of true positives is noted as *TP* in **Table 3** along with *FP* for false positives, *TN* for true negatives, and *FN* for false negatives. The framework recognizes sensitivity and specificity as fundamental analytical metrics because they enable the detection of diseased crops while correctly identifying healthy crops through their evaluation process.

## 4 Results

### 4.1 Experiments setting

Before presenting the experimental results, it is essential to outline the parameter configurations applied to each optimization algorithm and baseline model within this study. To ensure fair and reproducible comparisons, all metaheuristic optimizers were initialized using consistent global settings for population size, iteration count, and the number of independent runs. Additionally, each algorithm retained its standard control parameters as reported in the literature, unless otherwise specified. **Table 4** summarizes the specific hyperparameters used for Particle Swarm Optimization (PSO), Grey Wolf Optimizer (GWO), Genetic Algorithm (GA), and the proposed Puma Optimization (PO) method, which was applied to optimize the learning behavior of the Restricted Boltzmann Machine (RBM). These configurations provide the computational foundation for the comparative performance evaluation described in the subsequent sections.

**Table 4:** Parameters and settings for all algorithms used in the study.

Algorithm	Parameter	Value
All Algorithms	Population size	30
	Number of iterations	500
	Number of runs	30
PSO	Inertia (Wmax, Wmin)	[0.9, 0.6]
	Acceleration constants (C1, C2)	[2, 2]
GWO	*a*	2 to 0
GA	Mutation probability	0.05
	Crossover	0.02
	Fireflies	10
PO	PF1	0.5
	PF2	0.5
	PF3	0.3
	U	0.2
	L	0.9 (for F function), 0.7 (for C function)
	*a*	2 (for F function), 1 (for C function)

The computational environment consisted of a 64-bit Ubuntu 22.04 LTS system equipped with an Intel Core i7-11800H CPU @ 2.30GHz and 32 GB of RAM. All experiments used Python 3.10 with key dependencies, including NumPy, Pandas, Scikit-learn, Matplotlib, and PyTorch (version 1.13).

The RBM model was implemented using PyTorch and trained with the following hyperparameters: 50 epochs, batch size of 16, and a learning rate of 0.01. The optimizer was Adam, and training employed an L2 weight decay of 1e-4 to prevent overfitting. A dropout rate of 0.3 was applied to the visible layer during training. Early stopping was used with a patience threshold of 5 epochs based on validation loss.

To further improve robustness and minimize overfitting risk, a sparsity constraint was added to the RBM hidden layer, with a target sparsity value of 0.2. This encouraged sparse hidden representations, enhancing generalization under limited data conditions. Dropout was also applied not only at the visible layer but between subsequent dense layers with a variable rate ranging from 0.2 to 0.5 depending on validation performance.

The training strategy adopted PO (Puma Optimization) as the primary approach for hyperparameter tuning and RBM weight updates. By replacing conventional gradient-based methods, PO allowed dynamic exploration of the hyperparameter space, enabling convergence to robust configurations. The parameters tuned included the number of hidden units (range: 32 to 128), learning rate (range: 0.001 to 0.01), L2 regularization coefficient (range: $10^{-4}$ to $10^{-2}$ ), and sparsity target.

Cross-validation was conducted using a stratified 10-fold scheme to ensure class balance in each fold, enhancing the statistical reliability of performance estimates. In addition, nested cross-validation (5×2 folds) was applied for hyperparameter tuning to mitigate the risk of data leakage and overfitting during model selection.

Model performance was evaluated using stratified 5-fold cross-validation to ensure robust estimation on the small dataset. The original data was partitioned into 60% training, 20% validation, and 20% testing splits within each fold. Stratification preserved the proportion of disease and non-disease instances in all subsets.

**Figure 7:**
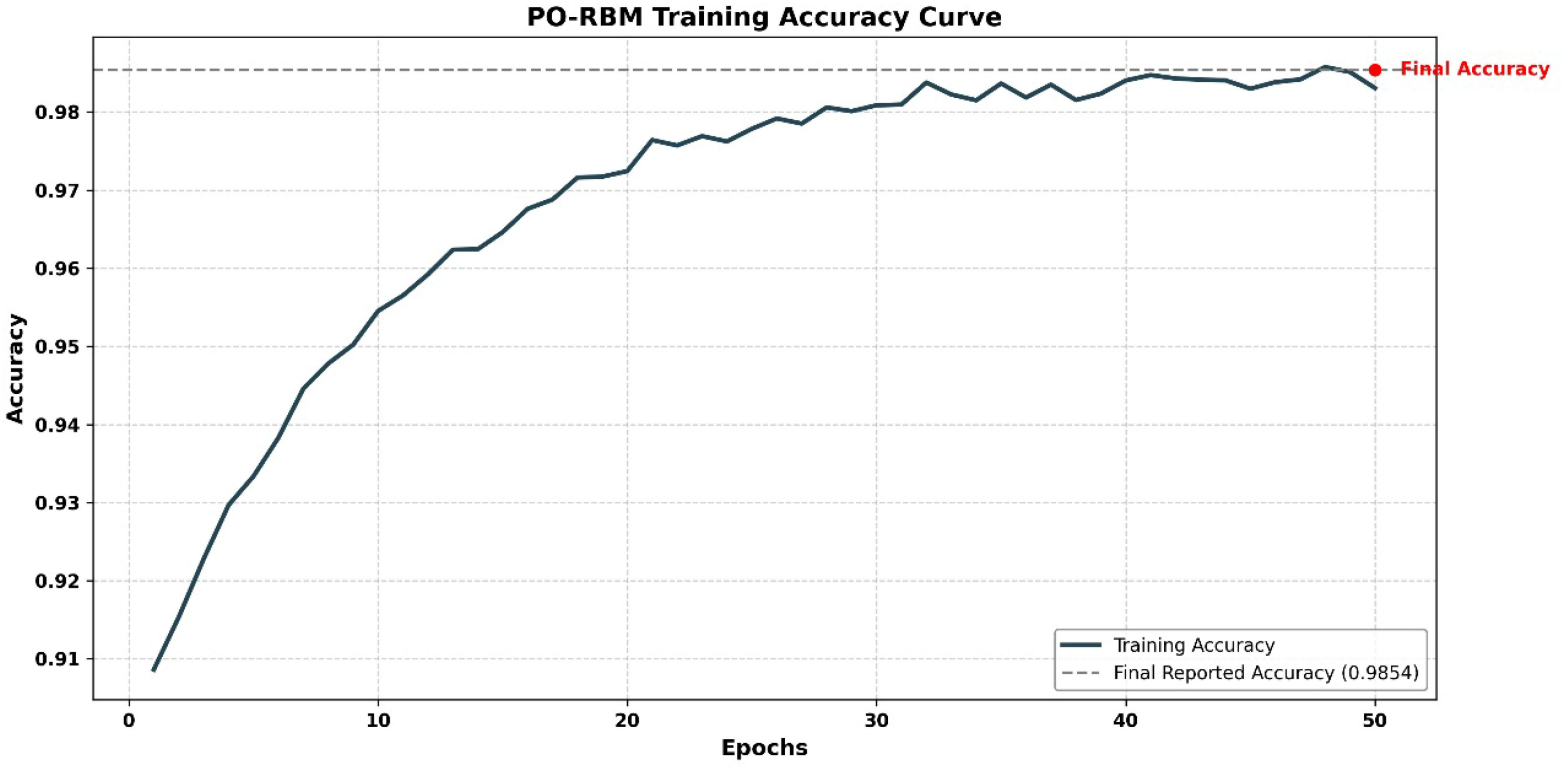
RBM training loss curve showing convergence over 50 epochs.

To confirm convergence and model stability, a training loss curve is presented in **Figure 7**, illustrating the progression of loss across epochs for the best-performing fold.

### 4.2 Baseline model performance

A comparative evaluation was conducted using five baseline classifiers: K-Nearest Neighbors (KNN), Random Forest (RF), XGBoost, Multi-Layer Perceptron (MLP), and an unoptimized Restricted Boltzmann Machine (RBM). As reported in **Table 5**, the RBM achieved the highest accuracy (94.77%) and F1-score (0.9506), demonstrating strong capability in modeling the nonlinear, high-dimensional relationships present in the agroecological dataset. Among the traditional models, XGBoost and RF performed competitively with favorable precision and specificity while requiring less computational time than RBM and MLP.

**Table 5:** Baseline classification performance of various models.

Models	Accuracy	Sensitivity (TRP)	Specificity (TNP)	PPV	NPV	FScore	Time (s)
KNN	0.9093	0.9264	0.8724	0.8458	0.9332	0.9401	217.564
RF	0.9175	0.9320	0.8870	0.8618	0.9386	0.9452	201.435
MLP	0.9195	0.9260	0.9123	0.9187	0.9231	0.9201	195.963
XGBoost	0.9271	0.9301	0.9229	0.9022	0.9376	0.9452	180.761
RBM	0.9477	0.9519	0.9429	0.9458	0.9506	0.9493	165.974

### 4.3 Optimized model comparison

The predictive performance of the Restricted Boltzmann Machine (RBM) was further improved through metaheuristic optimization using four algorithms: Particle Swarm Optimization (PSO), Grey Wolf Optimizer (GWO), Genetic Algorithm (GA), and the proposed Puma Optimization (PO). As shown in **Table 6**, PO-RBM achieved the highest classification accuracy (98.54%), as well as superior sensitivity, specificity, and F1-score compared to its counterparts. Additionally, PO-RBM demonstrated notable computational efficiency, completing training in less time (63.94 s) than PSO-RBM (97.54 s), GWO-RBM (104.44 s), and GA-RBM (118.77 s), due to its dynamic phase-switching mechanism that accelerates convergence without sacrificing performance.

**Table 6:** Comparison of the proposed PO-RBM model with other optimized RBM variants.

Models	Accuracy	Sensitivity (TRP)	Specificity (TNP)	PPV	NPV	FScore	Time (s)
PO-RBM	0.9854	0.9889	0.9823	0.9901	0.9845	0.9801	63.943
PSO-RBM	0.9699	0.9707	0.9691	0.9714	0.9695	0.9684	97.542
GWO-RBM	0.9637	0.9637	0.9638	0.9638	0.9637	0.9637	104.437
GA-RBM	0.9538	0.9525	0.9551	0.9526	0.9538	0.9550	118.765

**Figure 8:**
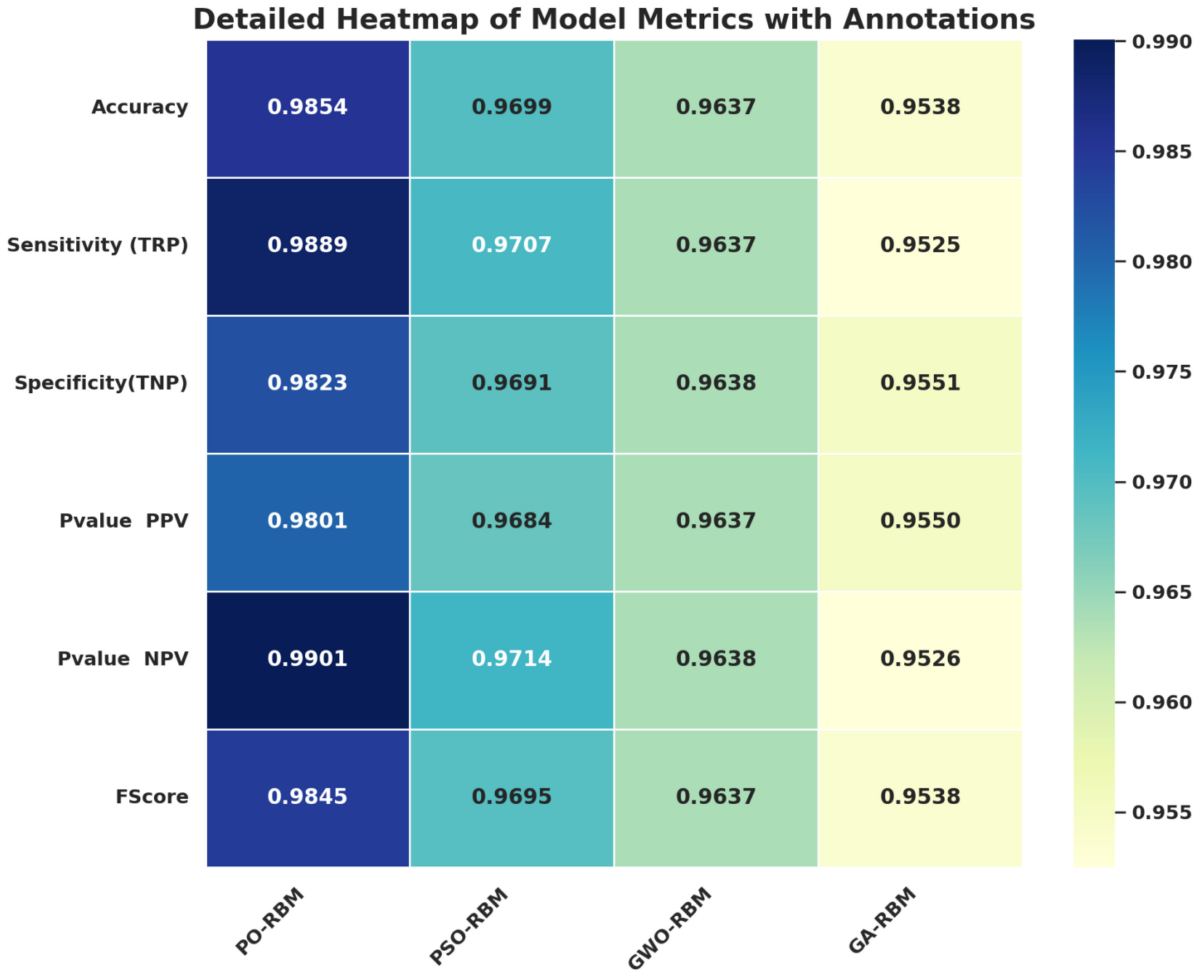
Heatmap of performance metrics across optimized RBM variants. PO-RBM consistently ranks highest.

A visual exploration of the results supports further statistical analysis. **Figure 8** presents a metric-bymodel heatmap, highlighting PO-RBM’s dominance across accuracy, sensitivity, specificity, PPV, NPV, and F-score.

**Figure 9:**
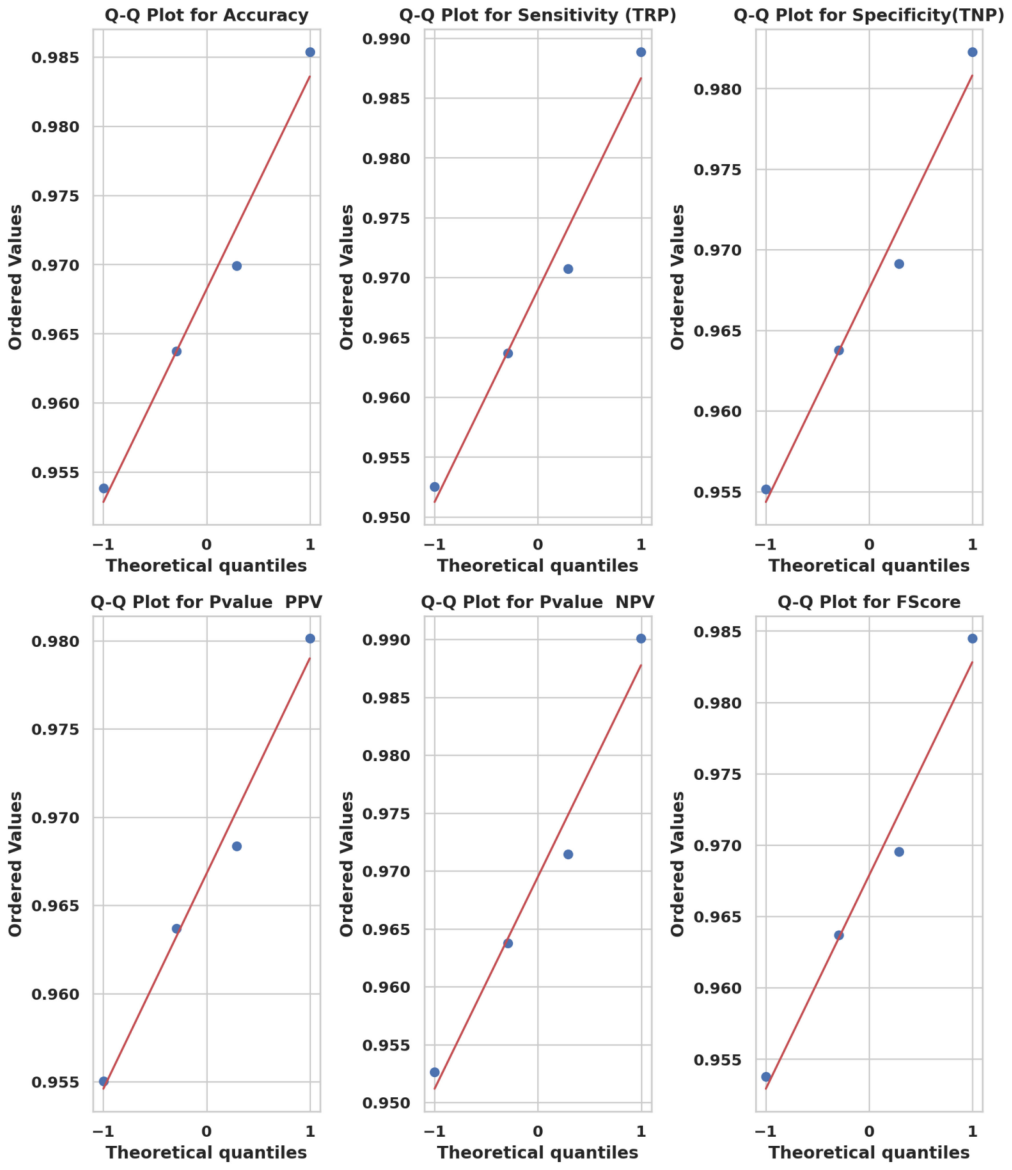
Q-Q plots of model metric distributions showing conformity to theoretical normal distributions.

**Figure 10:**
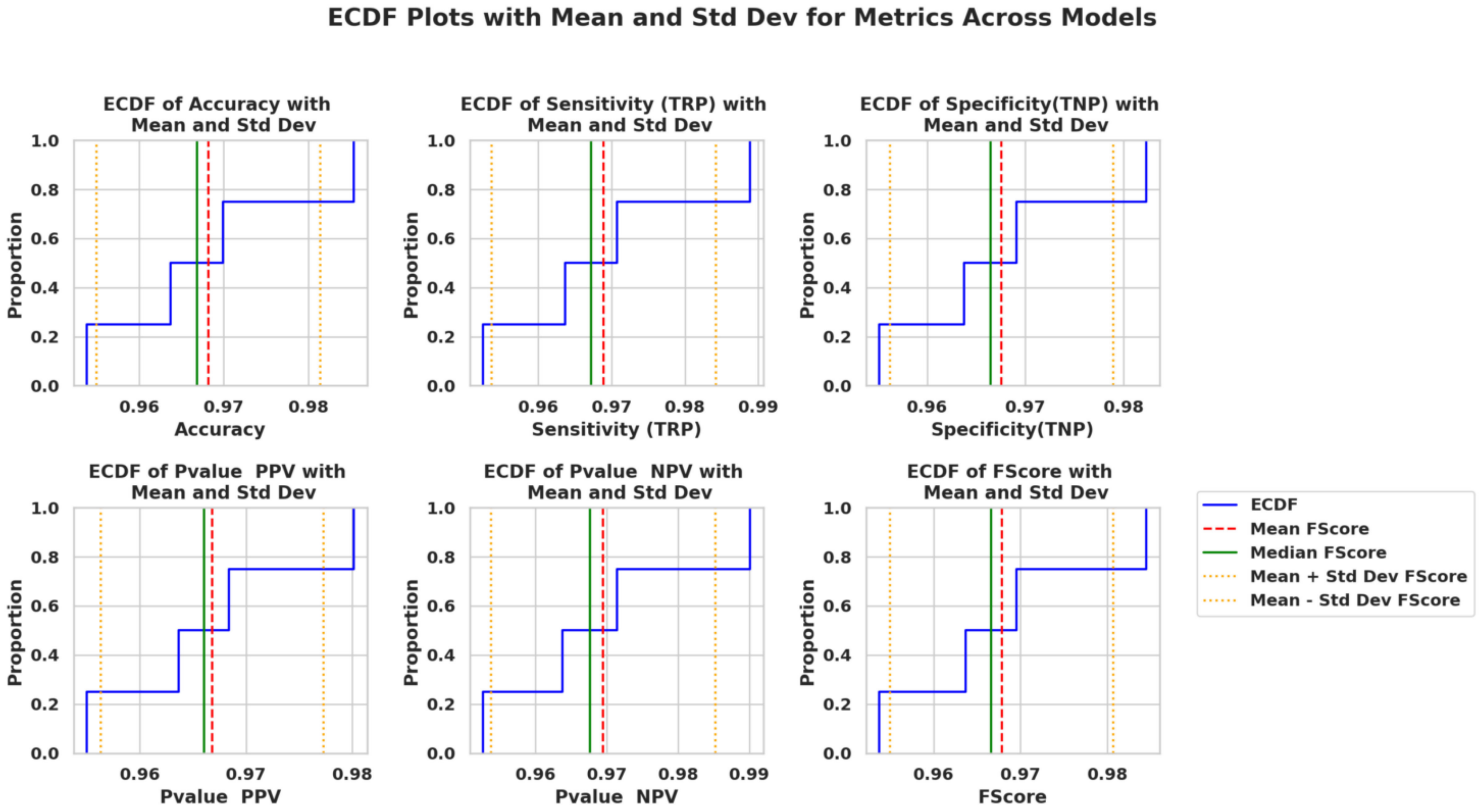
Empirical CDF (ECDF) metrics plots across models, including confidence bands and standard deviation margins.

Q-Q plots in **Figure 9** confirm that the performance scores across all models follow normality, validating the use of parametric tests. Similarly, ECDF plots in **Figure 10** demonstrate how PO-RBM maintains a high median and low variance for each performance metric.

From an ecological standpoint, these performance outcomes are highly beneficial. The model’s high sensitivity supports early-stage disease detection—crucial for minimizing pesticide reliance—while its high specificity helps prevent misclassification of healthy crops, thereby conserving resources and promoting sustainable field practices.

### 4.4 Statistical significance testing

Statistical significance testing was conducted using both parametric and non-parametric approaches to validate the observed performance differences among the optimized RBM variants. An analysis of variance (ANOVA) was applied to assess the overall variation in model accuracy across PO-RBM, PSO-RBM, GWO-RBM, and GA-RBM. The results, presented in **Table 7**, reveal a highly significant difference among the models (F(3, 36) = 73.13, p <0.0001), supporting the hypothesis that at least one model significantly outperforms the others.

**Table 7:** ANOVA test results for model comparisons.

ANOVA Table	SS	DF	MS	F (DFn, DFd)/P value
Treatment (between columns)	0.00546	3	0.00182	F(3, 36) = 73.13, P <0.0001
Residual (within columns)	0.0008959	36	0.00002489	
Total	0.006356	39		

A Wilcoxon signed-rank test was performed to confirm further the superiority of PO-RBM over the other optimization-enhanced RBM models. As summarized in **Table 8**, the PO-RBM achieved statistically significant improvements (p <0.002) over PSO-RBM, GWO-RBM, and GA-RBM across repeated runs. These results confirm that the performance advantages of PO-RBM are empirical and statistically robust.

**Table 8:** Wilcoxon signed-rank test results comparing PO-RBM with other models.

	PO-RBM	PSO-RBM	GWO-RBM	GA-RBM
Theoretical median	0	0	0	0
Actual median	0.9854	0.9699	0.9637	0.9538
Number of values	10	10	10	10
Wilcoxon Signed Rank Test				
Sum of signed ranks (W)	55	55	55	55
Sum of positive ranks	55	55	55	55
Sum of negative ranks	0	0	0	0
P value (two tailed)	0.002	0.002	0.002	0.002
Exact or estimate?	Exact	Exact	Exact	Exact
P value summary	**	**	**	**
Significant (alpha=0.05)?	Yes	Yes	Yes	Yes
How big is the discrepancy?				
Discrepancy	0.9854	0.9699	0.9637	0.9538

The * symbol expresses his values of the p value.

**Figure 11:**
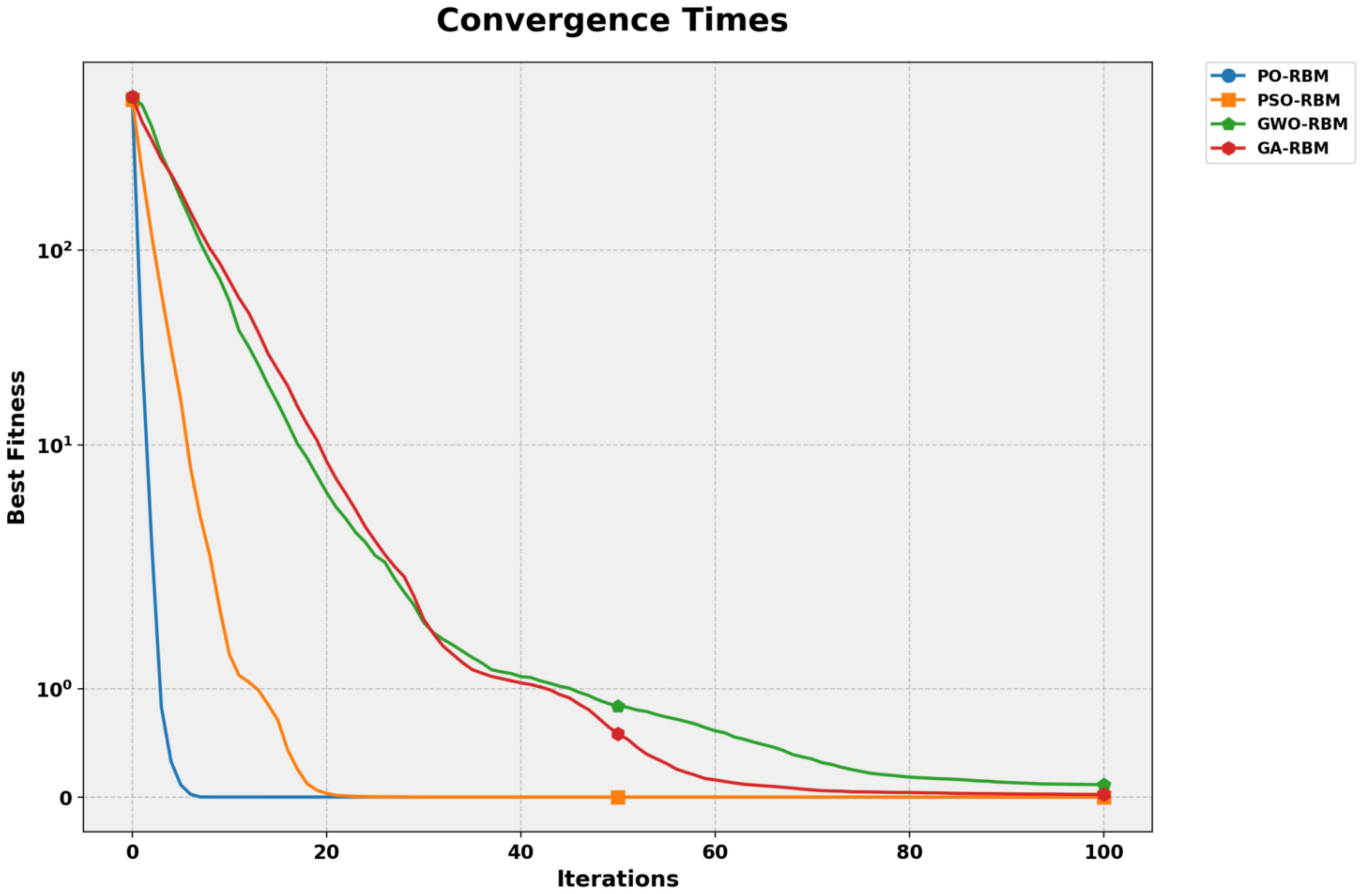
Convergence times for RBM optimization using PO, PSO, GWO, and GA. PO converges fastest with minimal oscillation.

A convergence analysis was performed for each optimization algorithm to illustrate training behavior further. As shown in **Figure 11**, PO-RBM reaches a stable solution significantly faster than the alternative metaheuristics, achieving convergence in fewer than 15 iterations. In contrast, PSO-RBM and GA-RBM required over 40 iterations for similar stabilization, with GWO-RBM exhibiting the slowest convergence trend.

**Figure 12:**
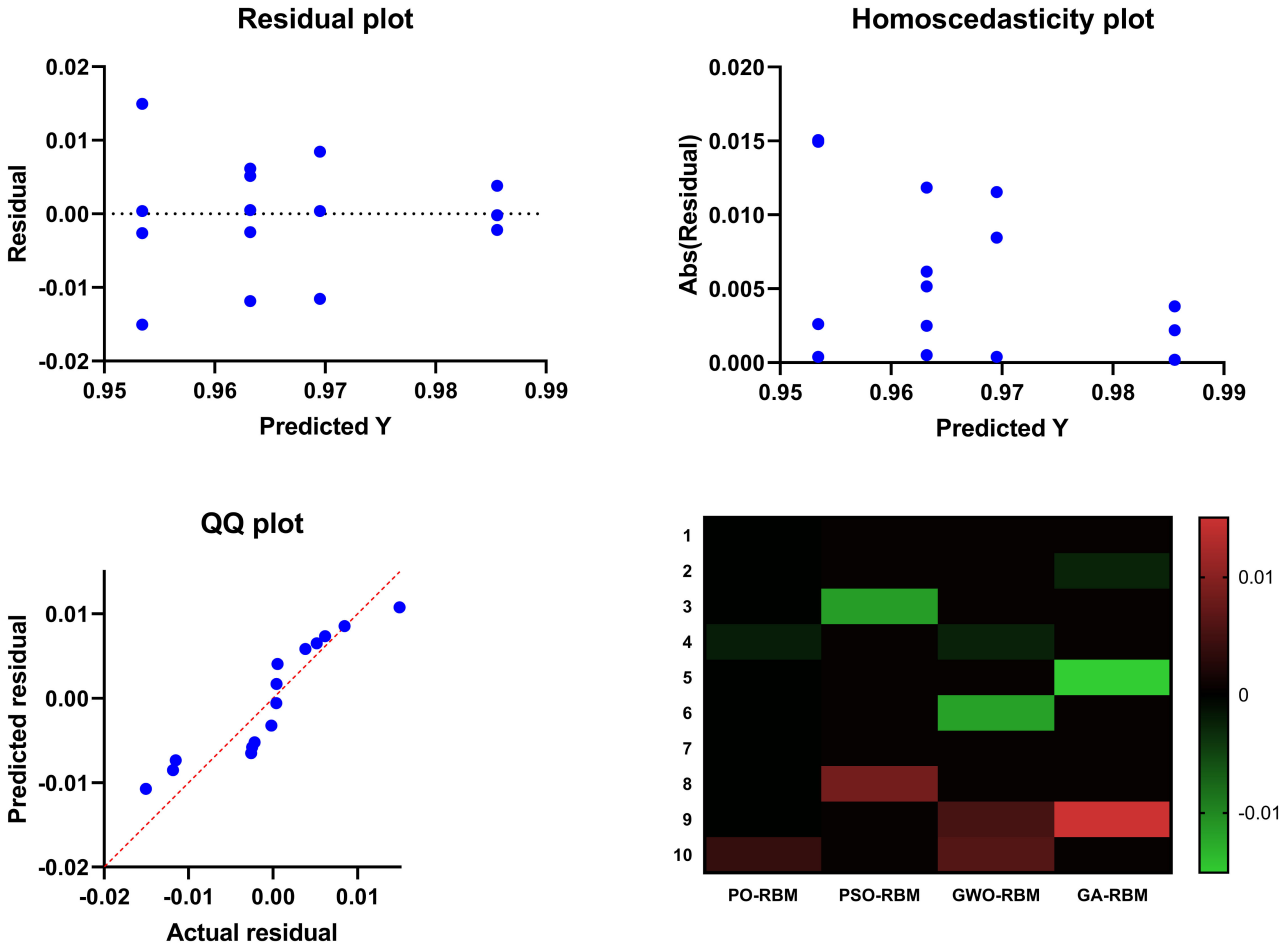
Summary visual comparison including scatter distribution, interquartile dispersion, and intermetric correlation for all models.

**Figure 13:**
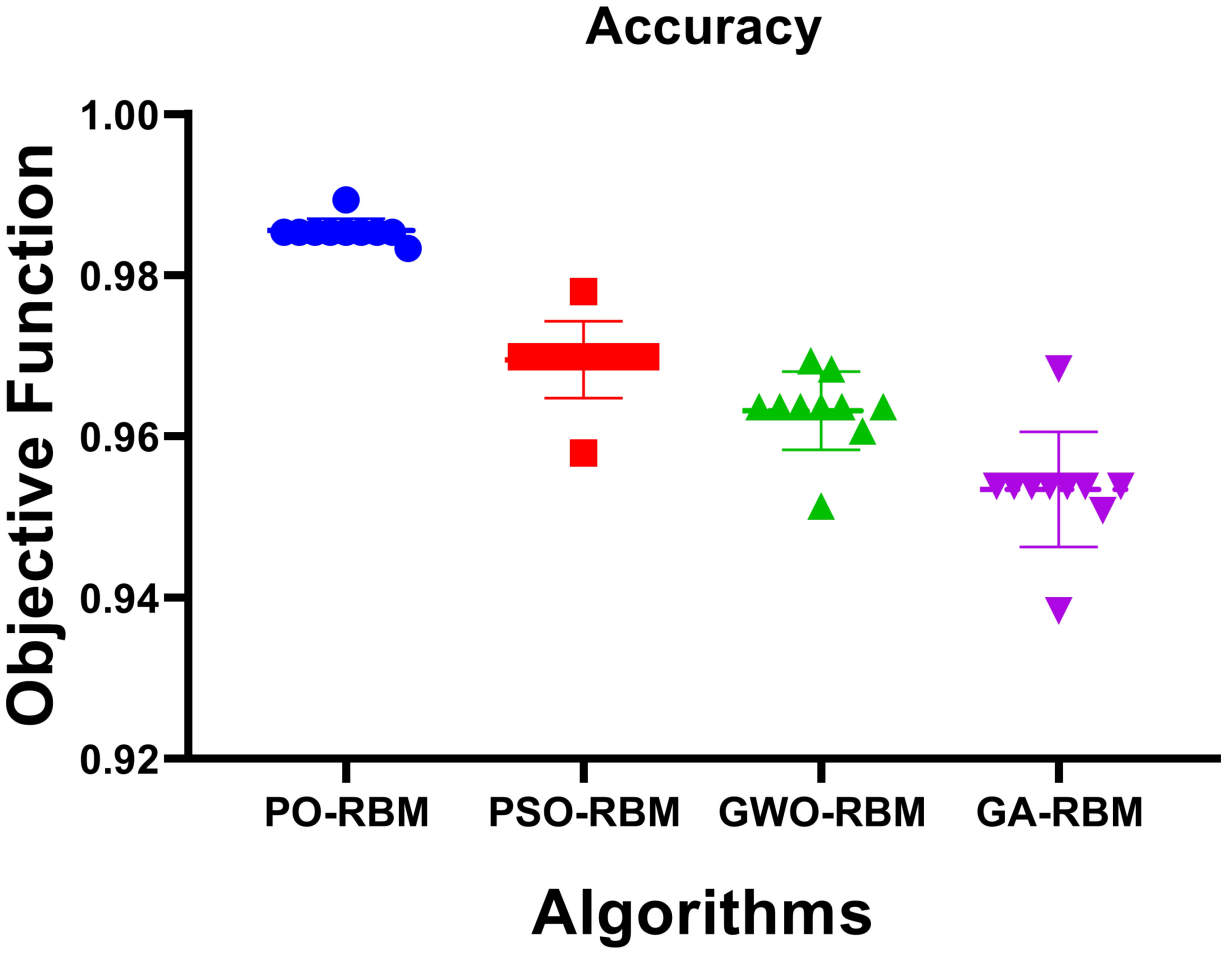
Boxplot representation of accuracy, sensitivity, specificity, and F-score, highlighting metric stability across models.

**Figures 12**, **13** provide visual confirmation of PO-RBM’s performance consistency, including dispersion, clustering, and overall metric reliability under ecological variability.

This advantage is complemented by the computational cost evaluation in **Table 6**, where PO-RBM achieved the lowest training time (63.94 seconds). The observed performance gain is attributed to PO’s dynamic phase-switching mechanism, accelerating convergence while maintaining high solution quality.

## 5 Discussion

Sustainable crop health management requires predictive technologies during climate change uncertainty and ecological stress, which must maintain accuracy and complex agroecosystem comprehension. The research presents a unified model agroecosystem for this need, which unites Restricted Boltzmann Machines (RBMs) representational capabilities with Puma Optimization (PO) algorithm adaptive capabilities. The system stands out because it evaluates potato diseases through multiple data types, including yield data, microbial levels, and environmental conditions, which function dynamically intertwined.

This strategy’s effectiveness produces hidden associations that traditional method frameworks would not discover. By incorporating copula-based dependency modeling before machine learning processes, the researchers improve feature alignment while respecting the probabilistic nature of ecological data that shows nonuniform and nonindependent interaction patterns. This complex model structure enables the system to explore challenging high-dimensional input data focusing on ecological interpretability, which many predictive agriculture systems currently sacrifice.

In particular, the copula-RBM combination improves ecological interpretability by explicitly modeling nonlinear dependencies between agronomic, microbial, and environmental features. This enables domain experts to identify how specific conditions—such as shifts in clade diversity or nutrient stress—jointly influence disease outcomes. The resulting probabilistic embeddings are predictive and transparent, facilitating agronomic decision-making grounded in meaningful variable interactions rather than opaque black-box outputs.

For example, the model analysis showed that high values of cladediv0.35 (a microbial clade diversity index) were strongly associated with reduced disease risk when nutrient availability was simultaneously high, as indicated by nutrients scores above the dataset median. Conversely, when asvdiv (amplicon sequence variant diversity) values dropped below a critical threshold (0.4), the RBM hidden layer activation patterns shifted toward the diseased class, especially under nutrient-poor conditions. These patterns were visualized via the RBM’s weight matrices and hidden node activations, confirming how combinations of microbial diversity and soil nutrient stress act synergistically in predicting disease onset. Such examples demonstrate that the model’s internal representations retain biological relevance and are interpretable by agronomic researchers.

Comparative evaluation with benchmark models—including KNN, RF, MLP, and XGBoost—demonstrated the RBM’s ability to capture complex, high-dimensional interactions relevant to agroecological systems. The unoptimized RBM outperformed these classifiers in accuracy and F1-score, and its performance was significantly elevated through PO-based optimization.

The robustness capabilities of the PO-RBM architecture become prominent because it maintains steadiness under various complex field settings. Agricultural information is characterized by noise and irregular patterns resulting from unmanageable environmental elements such as changing climate, varying soil conditions, and local climatic patterns. PO algorithm employs adaptive phase-switching to adjust its exploratory and exploitative activities, thus preventing premature convergence while achieving optimal model configurations across several performance landscapes. The improved prediction accuracy, along with trustworthy deployments, result from this system.

In addition to performance gains, the PO-RBM achieved faster convergence and lower training times than PSO-, GWO-, and GA-optimized RBMs, indicating computational advantages critical for real-world deployment. These findings are further supported by visual and statistical evidence, including ANOVA and Wilcoxon signed-rank testing, which confirm the significance of PO-RBM’s superiority (p <0.002).

While PO-RBM demonstrates strong theoretical performance, its operational potential in agricultural assessment remains a projection until validated in field trials. The model’s multi-modal data integration and probabilistic design align well with decision-support needs in integrated pest management (IPM), suggesting promise for eventual deployment. IPM strategies provide an optimal framework to embed the PO-RBM for delivering precise data-driven responses at different geographical scales. DiThis model could trigger Real-time disease alerts computational engine using this m, UAV imagery, and satellitederived indices are integrated. Such tools may enhance agricultural sustainability and support low-resource environments by reducing manual scouting and pesticide overuse, pending future empirical validation.

The system could be deployed as a modular service integrated with mobile apps, edge devices, or web platforms linked to on-farm IoT sensors and UAV-based imaging systems. For instance, soil nutrient and microbial sensor inputs could be streamed into the PO-RBM engine for real-time disease risk assessment. At the same time, mobile interfaces could notify farmers of actionable alerts. The architecture’s flexibility supports scalable deployment across regions with variable infrastructure readiness.

This study has its limitations as any other modeling approach which derives its foundation from real-world data. The available dataset introduces biological diversity but provides narrow-period information and limited widespread use possibilities. The development of diseases as biological entities propagates from weeks to months while affected by various organism and non-organismal environmental systems. Extending the model to process time-series information would enable its application to multi-crop environments and remote sensing capabilities for future scope enhancement. Expanding the system to detect disease severity rather than make binary classifications would provide researchers with more precise dynamics to direct precise disease management practices.

The PO-RBM framework operates beyond the machine learning model status. It provides an expandable system that unites ecological expertise with probabilistic methods alongside bio-inspired optimization into plant health analysis systems. This research contributes to creating innovative, sustainable agricultural tools by connecting technological innovation with environmental protection, thus developing forecasting systems that match the adaptability of their target ecosystems.

## 6 Study limitations

While the proposed PO-RBM framework demonstrates promising results for potato disease classification, several limitations must be acknowledged to contextualize its current scope and guide future improvements.

### 6.1 Dataset size and sampling scope

The study was based on a relatively small dataset of 52 samples collected across 26 field sites and two experimental seasons. Although stratified sampling ensured a balanced representation of disease and non-disease cases, the limited sample size may constrain the model’s robustness and ability to capture rare or region-specific disease patterns.

### 6.2 Temporal limitations

The dataset represents a single snapshot per field-season pair, lacking the continuous time-series data necessary to model disease progression or seasonality. This restricts the current system’s utility for forecasting temporal dynamics such as early-stage infection detection or long-term pest risk.

### 6.3 Crop specificity

The model was trained and validated exclusively on potato-specific agronomic and microbial indicators. While the architecture is generalizable in theory, applying it to other crops would require extensive retraining with domain-specific ecological inputs, as disease mechanisms vary significantly across plant species.

### 6.4 Field validation and real-world deployment

No field trials were conducted to validate predictions under operational farm conditions. As a result, practical deployment considerations such as integration latency, sensor calibration variance, or farmer usability feedback remain unexplored at this stage.

### 6.5 Computational complexity

While the PO-RBM offers interpretability and high accuracy, its training pipeline—including copula transformations and metaheuristic tuning—requires more computational resources than traditional classifiers. This may limit its use on resource-constrained edge devices without further optimization.

These limitations are significant to consider when interpreting the study’s findings. They also offer clear directions for ongoing research, including dataset expansion, time-series modeling, cross-crop generalization, and real-world pilot testing.

## 7 Conclusion and future work

This study presents a hybrid machine learning framework for potato disease classification that combines copula-based dependency modeling with a Restricted Boltzmann Machine (RBM), further enhanced through Puma Optimization (PO). The proposed PO-RBM model detects complicated nonlinear relationships between agricultural and ecological parameters, from yield changes to microbial variations, and self-adjusts its inner components for peak identification accuracy. The framework moves toward technical excellence by combining probabilistic learning with adaptive metaheuristic optimization. It solves practical challenges that aim to decrease pesticide use while improving disease monitoring and IPM decision support.

Baseline evaluations demonstrated that the RBM outperforms conventional classifiers such as KNN, Random Forest, MLP, and XGBoost, particularly in capturing complex agronomic-microbial interactions. PO-based optimization further improved performance to 98.54% accuracy, with accompanying gains in sensitivity, specificity, and F1-score. These improvements were statistically validated using ANOVA and Wilcoxon signed-rank tests, while convergence and training-time analyses highlighted PO’s computational efficiency over other optimizers. These findings establish the PO-RBM framework as a viable, scalable solution for precision agriculture. In the near term, it may serve as the backbone for adaptive disease surveillance systems, especially within integrated pest management (IPM) programs.

Expert researchers will continue working on this framework to enhance its potential spread throughout various agricultural regions and climate zones. The early-warning platform will reach full operational capacity when the system integrates time-based and geography-based data layers, such as temporal satellite imagery and IoT-based climate monitoring systems. One future extension includes incorporating temporal dependencies using recurrent architectures like time-aware RBMs or Long Short-Term Memory (LSTM) networks to support dynamic disease forecasting. Integrating high-resolution multispectral drone data with microbiome shifts could improve early detection precision.

Policymakers from a version of the PO-RBM framework designed for stakeholder-specific thresholds in sustainable interventions. Local government policy policymakers ‘ predictive designing ecological understanding by extending the present architectural approach to multi-class disease severity and developing hybrid networks combining convolutional and recurrent systems. PO-RBM tools will maintain their primary function in precision farming as they develop to establish resilient agricultural systems that preserve resources and respect the environment.

## Data Availability

The original contributions presented in the study are included in the article/supplementary material. Further inquiries can be directed to the corresponding authors.

## References

[B1] Abdollahzadeh B. Khodadadi N. Barshandeh S. Trojovský P. Gharehchopogh F. S. El-kenawy E.-S. M.. (2024). Puma optimizer (po): a novel metaheuristic optimization algorithm and its application in machine learning. Cluster Computing 27, 5235–5283. doi: 10.1007/s10586-023-04221-5

[B2] Agarwal M. Sinha A. Gupta S. K. Mishra D. Mishra R. (2020). “Potato crop disease classification using convolutional neural network,” in Smart Systems and IoT: Innovations in Computing. Eds. Somani A. K. Shekhawat R. S. Mundra A. Srivastava S. Verma V. K. (Singapore: Springer), 391–400. doi: 10.1007/978-981-13-8406-637

[B3] Ashourloo D. Shahrabi H. S. Azadbakht M. Rad A. M. Aghighi H. Radiom S. (2020). A novel method for automatic potato mapping using time series of sentinel-2 images. Comput. Electron. Agric. 175, 105583. doi: 10.1016/j.compag.2020.105583

[B4] Ayilara M. S. Adeleke B. S. Akinola S. A. Fayose C. A. Adeyemi U. T. Gbadegesin L. A.. (2023). Biopesticides as a promising alternative to synthetic pesticides: A case for microbial pesticides, phytopesticides, and nanobiopesticides. Front. Microbiol. 14. doi: 10.3389/fmicb.2023.1040901, PMID: 36876068 PMC9978502

[B5] Bienkowski D. Aitkenhead M. J. Lees A. K. Gallagher C. Neilson R. (2019). Detection and differentiation between potato (solanum tuberosum) diseases using calibration models trained with non-imaging spectrometry data. Comput. Electron. Agric. 167, 105056. doi: 10.1016/j.compag.2019.105056

[B6] Breiman L. (2001). Random forests. Mach. Learn. 45, 5–32. doi: 10.1023/A:1010933404324

[B7] Chauhan R. Prajapati A. Vaidya H. (2023). “Potato disease detection and recognition using deep learning,” in 2023 3rd International Conference on Innovative Sustainable Computational Technologies (CISCT). (Dehradun, India: IEEE) 1–6. doi: 10.1109/CISCT57197.2023.10351289

[B8] Chen T. Guestrin C. (2016). “Xgboost: A scalable tree boosting system,” in Proceedings of the KDD '16: The 22nd ACM SIGKDD International Conference on Knowledge Discovery and Data Mining San Francisco California USA (New York, NY, USA: Association for Computing Machinery). 785–794. doi: 10.1145/2939672.2939785

[B9] Cheng D. Zhao Z. Jiang F. (2024). Rice diseases identification method based on improved yolov7-tiny. Agriculture 14, 709–709. doi: 10.3390/agriculture14050709

[B10] Durai S. Sujithra T. Iqbal M. M. (2023). “Image classification for potato plant leaf disease detection using deep learning,” in 2023 International Conference on Sustainable Computing and Smart Systems (ICSCSS). (Coimbatore, India: IEEE) 154–158. doi: 10.1109/ICSCSS57650.2023.10169446

[B11] Elbasi E. Zaki C. Topcu A. E. Abdelbaki W. Zreikat A. I. Cina E.. (2023). Crop prediction model using machine learning algorithms. Appl. Sci. 13, 9288–9288. doi: 10.3390/app13169288

[B12] Fischer A. Igel C. (2012). “An introduction to restricted boltzmann machines,” in Progress in Pattern Recognition, Image Analysis, Computer Vision, and Applications. (Berlin, Heidelberg: Springer), 7441. doi: 10.1007/978-3-642-33275-3_2

[B13] Gao J. Westergaard J. C. Sundmark E. H. R. Bagge M. Liljeroth E. Alexandersson E. (2021). Automatic late blight lesion recognition and severity quantification based on field imagery of diverse potato genotypes by deep learning. Knowledge-Based Syst. 214, 106723. doi: 10.1016/j.knosys.2020.106723

[B14] Gijsbers P. Pfisterer F. van Rijn J. N. Bischl B. Vanschoren J. (2021). Meta-learning for symbolic hyperparameter defaults. Association for Computing Machinery. 151–152. doi: 10.1145/3449726.3459532

[B15] Guo G. Wang H. Bell D. Bi Y. Greer K. (2003). “Knn model-based approach in classification,” in On The Move to Meaningful Internet Systems 2003: CoopIS, DOA, and ODBASE. Eds. Meersman R. Tari Z. Schmidt D. C. (Springer Berlin Heidelberg, Berlin, Heidelberg), 986–996.

[B16] Hou C. Zhuang J. Tang Y. He Y. Miao A. Huang H.. (2021). Recognition of early blight and late blight diseases on potato leaves based on graph cut segmentation. J. Agric. Food Res. 5, 100154. doi: 10.1016/j.jafr.2021.100154

[B17] Jha G. K. Velayudhan P. K. Bhatia A. Laishram C. Kumar D. Begho T.. (2024). Transitioning towards sustainable agriculture: analysing the factors and impact of adopting multiple sustainable inputs by paddy farmers in India. Front. Sustain. Food Syst. 8. doi: 10.3389/fsufs.2024.1447936

[B18] Ji Y. Sun L. Li Y. Li J. Liu S. Xie X.. (2019). Non-destructive classification of defective potatoes based on hyperspectral imaging and support vector machine. Infrared Phys. Technol. 99, 71–79. doi: 10.1016/j.infrared.2019.04.007

[B19] Joe H. (2014). Dependence Modeling with Copulas (New York: Chapman and Hall/CRC). doi: 10.1201/b17116

[B20] Kaur J. Parmar K. S. Singh S. (2023). Autoregressive models in environmental forecasting time series: a theoretical and application review. Environ. Sci. pollut. Res. 30, 19617–19641. doi: 10.1007/s11356-023-25148-9, PMID: 36648728 PMC9844203

[B21] Kennedy J. Eberhart R. (1995). “Particle swarm optimization,” in Proceedings of ICNN’95 International Conference on Neural Networks, (Perth, WA, Australia: IEEE) Vol. 4. 1942–1948. doi: 10.1109/ICNN.1995.488968

[B22] Mahum R. Munir H. Mughal Z.-U.-N. Awais M. Khan F. S. Saqlain M.. (2023). A novel framework for potato leaf disease detection using an efficient deep learning model. Hum. Ecol. Risk Assessment: Int. J. 29, 303–326. doi: 10.1080/10807039.2022

[B23] Marino S. Beauseroy P. Smolarz A. (2019). Weakly-supervised learning approach for potato defects segmentation. Eng. Appl. Artif. Intell. 85, 337–346. doi: 10.1016/j.engappai.2019.06.024

[B24] Markam S. S. Raj A. Kumar A. Khan M. L. (2024). Microbial biosurfactants: Green alternatives and sustainable solution for augmenting pesticide remediation and management of organic waste. Curr. Res. Microbial. Sci. 7, 100266–100266. doi: 10.1016/j.crmicr.2024.100266, PMID: 39257939 PMC11385824

[B25] Mirjalili S. Mirjalili S. M. Lewis A. (2014). Grey wolf optimizer. Adv. Eng. Software 69, 46–61. doi: 10.1016/j.advengsoft.2013.12.007

[B26] Ngugi H. N. Ezugwu A. E. Akinyelu A. A. Abualigah L. (2024). Revolutionizing crop disease detection with computational deep learning: a comprehensive review. Environ. Monit. Assess. 196. doi: 10.1007/s10661-024-12454-z, PMID: 38401024 PMC10894121

[B27] Okagu I. U. Okeke E. S. Ezeorba W. C. F. Ndefo J. C. Ezeorba T. P. C. (2023). Overhauling the ecotoxicological impact of synthetic pesticides using plants’ natural products: a focus on zanthoxylum metabolites. Environ. Sci. pollut. Res. 30, 67997–68021. doi: 10.1007/s11356-023-27258-w, PMID: 37148518 PMC10212812

[B28] Popescu M.-C. Balas V. Perescu-Popescu L. Mastorakis N. (2009). Multilayer perceptron and neural networks. WSEAS Trans. Circuits Syst. 8.

[B29] Pradhan B. Sameen M. I. Al-Najjar H. A. H. Sheng D. Alamri A. Park H. (2021). A metalearning approach of optimisation for spatial prediction of landslides. Remote Sens. 13, 4521–4521. doi: 10.3390/rs13224521

[B30] Pukrongta N. Taparugssanagorn A. Sangpradit K. (2024). Enhancing crop yield predictions with pensemble 4: Iot and ml-driven for precision agriculture. Appl. Sci. 14, 3313–3313. doi: 10.3390/app14083313

[B31] Qu B. Xiao Z. Upadhyay A. Luo Y. (2024). Perspectives on sustainable food production system: Characteristics and green technologies. J. Agric. Food Res. 15, 100988–100988. doi: 10.1016/j.jafr.2024.100988

[B32] Reeves C. (2010). “Genetic algorithms,” in Handbook of Metaheuristics, 146, 109–139. doi: 10.1007/978-1-4419-1665-55

[B33] Rodríguez J. Lizarazo I. Prieto F. Angulo-Morales V. (2021). Assessment of potato late blight from uav-based multispectral imagery. Comput. Electron. Agric. 184, 106061. doi: 10.1016/j.compag.2021.106061

[B34] Shaheed K. Qureshi I. Abbas F. Jabbar S. Abbas Q. Ahmad H.. (2023). Efficientrmt-net—an efficient resnet-50 and vision transformers approach for classifying potato plant leaf diseases. Sensors 23, 9516–9516. doi: 10.3390/s23239516, PMID: 38067888 PMC10708852

[B35] Srivastava A. Saini P. K. Kumar K. Tiwari S. Garg N. (2024). “Potato leaf disease detection using dense net-cnn,” in 2024 2nd International Conference on Intelligent Data Communication Technologies and Internet of Things (IDCIoT). (Bengaluru, India: IEEE) 648–653. doi: 10.1109/IDCIoT59759.2024.10467587

[B36] Vázquez-Ramírez S. Torres-Ruiz M. Quintero R. Chui K. T. Sanchéz-Mejorada C. G. (2023). An analysis of climate change based on machine learning and an endoreversible model. Mathematics 11, 3060–3060. doi: 10.3390/math11143060

[B37] Xing Y. Wang X. (2024). Precise application of water and fertilizer to crops: challenges and opportunities. Front. Plant Sci. 15. doi: 10.3389/fpls.2024.1444560, PMID: 39711591 PMC11659019

[B38] Yang L. Yu X. Zhang S. Zhang H. Xu S. Long H.. (2023). Stacking-based and improved convolutional neural network: a new approach in rice leaf disease identification. Front. Plant Sci. 14. doi: 10.3389/fpls.2023.1165940, PMID: 37346133 PMC10279891

[B39] Yang Y. Zhao X. Huang M. Wang X. Zhu Q. (2021). Multispectral image based germination detection of potato by using supervised multiple threshold segmentation model and canny edge detector. Comput. Electron. Agric. 182, 106041. doi: 10.1016/j.compag.2021.106041

[B40] Zhang W. Zhu Q. Huang M. Guo Y. Qin J. (2019). Detection and classification of potato defects using multispectral imaging system based on single shot method. Food Analytical Methods 12, 2920–2929. doi: 10.1007/s12161-019-01654-w

